# Fractional-Order Chaotic Memory with Wideband Constant Phase Elements

**DOI:** 10.3390/e22040422

**Published:** 2020-04-09

**Authors:** Jiri Petrzela

**Affiliations:** Department of Radio Electronics, Faculty of Electronical Engineering and Communications, Brno University of Technology, 616 00 Brno, Czech Republic; petrzelj@feec.vutbr.cz

**Keywords:** approximate entropy, admittance function synthesis, constant phase element, chaotic oscillator, fractional-order, frequency response, ternary memory, zeroes and poles

## Abstract

This paper provides readers with three partial results that are mutually connected. Firstly, the gallery of the so-called constant phase elements (CPE) dedicated for the wideband applications is presented. CPEs are calculated for 9° (decimal orders) and 10° phase steps including ¼, ½, and ¾ orders, which are the most used mathematical orders between zero and one in practice. For each phase shift, all necessary numerical values to design fully passive RC ladder two-terminal circuits are provided. Individual CPEs are easily distinguishable because of a very high accuracy; maximal phase error is less than 1.5° in wide frequency range beginning with 3 Hz and ending with 1 MHz. Secondly, dynamics of ternary memory composed by a series connection of two resonant tunneling diodes is investigated and, consequently, a robust chaotic behavior is discovered and reported. Finally, CPEs are directly used for realization of fractional-order (FO) ternary memory as lumped chaotic oscillator. Existence of structurally stable strange attractors for different orders is proved, both by numerical analyzed and experimental measurement.

## 1. Introduction

Recently, utilization of FO circuit elements in the analog signal processing applications attracts increasing interest among researchers and especially circuit design engineers [1]. Despite significant manufacturing efforts, circuit elements characterized by a FO network function close-enough to ideal are still not commercially available. Thus, behavior of FO two-terminal or two-port device should be approximated, either in time domain or, more commonly, in frequency domain. In the latter case, we must construct a robust circuit with the constant phase shift between response (voltage or current) and driving force (voltage or current) from DC to infinite frequency. Obviously, a circuit cannot satisfy such requirement. Thus, approximation of CPE is valid only in some limited frequency range predefined by application. Concrete value of a phase shift depends on mathematical order of CPE. In practice, CPEs are primarily constructed as two-terminal devices and mostly for the non-integer orders between zero and one; to replace standard capacitor with the so-called fractal capacitor. In this case, phase shift between current and voltage is 90*α*°, where *α*∈(0, 1) is mathematical order of designed CPE. If speaking in terms of module frequency response, admittance linearly increases (in logarithmic horizontal scale), namely with slope 20*α* dB per frequency decade. Besides fractal capacitor, we can find FO integrator, i.e., two-port where voltage transfer function has FO character. Higher non-integer orders can be implemented easily by a cascading two or more FO integrators. It is much more transparent than utilization of the general immittance converters to create FO immittance with an order higher than one. Some interesting structures of immittance converters capable to create arbitrary FO can be found in papers [2,3,4]. In addition, conventional topologies such as the general immittance converters by Antoniou and Riordan can be used; namely to construct non-integer immittance up to the fifth order. Few specific network topologies allow change of the order without reconfiguration or reconnection. For example, the circuit proposed in [5] simultaneously changes all time constants inside FO immittance via adjustment of the trans-conductance *g_m_* of the operational trans-conductance amplifiers that are controlled by external DC voltages. However, practical applicability is questionable due to nonlinear *g_m_* control, temperature changes of *g_m_*, and high sensitivities.

In the last decade, tens of papers deal with analysis of the conventional building blocks where standard accumulation element (capacitor, inductor) is replaced by FO equivalent. By doing this substitution, mathematical description turns into FO domain, i.e., mathematical model contains one or several FO ordinary differential equations. Such a transformation can, in some situations, result into advantageous properties or features of an “improved” building block. On the other hand, the replacement mentioned above can lead to analog functional blocks that exhibit worse or different behavior than anticipated. Let us briefly discuss a few examples from the area of FO frequency filters, FO oscillators, FO PID regulators, mathematical modeling using FO calculus, new applications, and others.

The first group of analog lumped electronic systems, where FO circuit elements were heavily tested, most likely covers frequency filters. Basic studies dealing with first order FO filters having single or coupled CPE can be found in pioneering works [6,7,8]. It can be shown that order-less-than-one band-pass and band-reject filters can be constructed [9,10]. Besides this advantage, substitution of standard capacitor by FO equivalent can change complex voltage transfer function so that the analyzed filter is of a different type. For example, FO all-pass filter, both first and higher order, cannot be created by the mentioned substitution [11]. In some papers, this problem has not been recognized [12]. Much more attention was paid on second order FO filters. Some research works are focused on the general properties of this class of analog building blocks [13,14]. Other studies are aimed at specific structures such as Sallen-Key and KHN filters [15], passive and active realizations of filters having a Butterworth-type of frequency response [16], low-pass filter with transfer zeroes [17], low-pass with electronically reconfigurable parameters [18], pseudo-differential all-pass filter [19], etc. A FO filter can be constructed by using field programmable analog array as well [20,21]. The above mentioned publications prove that FO filters can take some advantage over conventional integer-order (IO) equivalents. Unique properties of FO elements can be used to construct two-ports with properties that are unreachable by IO circuits. For example, in the case of IO two-ports, only natural multiples of 90° asymptotical phase shifts can be performed. However, generalization of the same network into FO domain removes this restriction. Two-ports having arbitrary starting and ending phase shift between response and driving force can be found in [22] for negative and [23] positive phase shift derivation with respect to frequency. A similar situation can be observed in the case of FO generators of harmonic waveforms. A very good cookbook dealing with the construction of oscillators with one or several FO two-terminal devices is provided in step-by-step manner in paper [24]. Similar as in filter theory, the contribution of many papers in this research field is based on a simple interchange of standard linear capacitor with FO equivalent. Thus, common network structures of harmonic oscillators are analyzed. For example, Colpitts [25] or Wien-bridge [26] oscillator topology already undergoes deep computer-aided analysis. Besides these studies, some “new” concepts were re-discovered. A harmonic oscillator with voltage buffers and operational trans-conductance amplifiers is subject of paper [27]. The problem of modeling of FO differential equations via lumped electronic circuits was also addressed in the recent publications. The design of a simple chaotic oscillator based on jerky dynamics with FO inductor is the topic of this paper [28]. Circuitry realization of a much more complex chaotic system can be found in [29], where FO memristor is employed. FO circuit elements found applications in feedback regulators and control. For example, passive ladder CPE provides better voltage regulation than integer-order circuit configuration, as demonstrated in [30]. PID regulators with FO integration and differentiator branch can provide smooth regulation of abrupt plant processes. A general study of this phenomenon can be found in study [31], while a complete practical design of a PID regulator is the subject of fundamental paper [32]. A different approach to regulation is shown in [33], where FO, ID, and combined ID control is associated with input impedances of two-terminal devices. A practical example, namely FO feedback control of dc motor or modeling of dc-dc-converter behavior, is described in works [34,35], respectively. A list of potential applications of FO elements mentioned above is by no means complete. Realization of FO systems still represents up-to-date topic addressed by circuit design engineers.

This paper is organized as follows. Section 2 describes, from a circuit synthesis point of view, various implementations of CPE approximants. Here we can find two-terminal RC and RL passive ladder structures. Individual networks are introduced without giving numerical values of the passive components. These are provided in Section 3 in a tabularized form and for all mathematical orders of CPE and two equivalent RC ladders. Section 4 demonstrates two fundamental transformations that can fit CPEs to specific situation: impedance matching, and frequency rescale. Section 5 shows numerical analysis of FO binary and ternary memory where conventional capacitors and designed CPEs are considered to be parasitic accumulation elements. This section also describes circuitry realization of analyzed memories including experimental confirmation of chaos via oscilloscope screenshots. Finally, discussion and concluding remarks are provided.

## 2. Design Methods Dedicated for CPE

As mentioned before, CPE is usually approximated in the frequency domain. It means that the higher order circuit having complex network function realizes CPE. To be more specific, this network function has several real negative zeroes and poles that alternates on frequency axis and this variation of zeroes and poles creates final ripple of phase frequency response. Each CPE is designed based on three input parameters: frequency range (depends on the future applications), maximal phase error (should be as small as possible) and complexity (each zero and pole pair needs to be implemented by additional sub-circuit). For given frequency interval, maximal phase difference between ideal and approximated CPE is inversely proportional to the circuit complexity, i.e., very accurate CPEs have a FO network function with many zeroes and poles.

In the case of proposed wideband CPEs, the frequency band begins at 3 Hz and finishes at 1 MHz. To preserve distinguishability between individual orders of CPEs, the maximal phase error needs to be lower than 1.5°. Having these two input parameters defined the resulting complexity as a sixth order network function. This general network function can be written in Laplace transform as

(1) $$F\left(s\right)=\frac{\sum_{k=0}^{8}a_{k}s^{k}}{\sum_{k=0}^{8}b_{k}s^{k}}=\frac{a_{8}}{b_{8}}\cdot \frac{\prod_{k=1}^{8}\left(s+2\pi f_{Zk}\right)}{\prod_{k=1}^{8}\left(s+2\pi f_{Pk}\right)},$$

where *s* is a complex frequency, *a_k_* and *b_k_* are real positive coefficients, and *z_k_* and *p_k_* are zeroes and poles of network function. Of course, if function (1) is at least of second order it can be further decomposed into biquadratic sub-sections. Zeroes and poles of a complex network function (1) are real, negative (it is system with minimal argument) and alternates equidistantly (in a logarithmic scale) on the frequency axis. This alternation creates final phase ripple around theoretical value given as 90°*α,* where *α* is a non-integer order of designed CPE. Phase ripple in degrees that can be expected in the case of CPE approximation is evident from formula

(2) $$\varphi \left(f\right)=\frac{180}{\pi }\cdot \left(\sum_{k=1}^{7}atan\frac{f}{f_{Zk}}-\sum_{k=1}^{7}atan\frac{f}{f_{Pk}}\right)\;.$$

**Figure 1:**
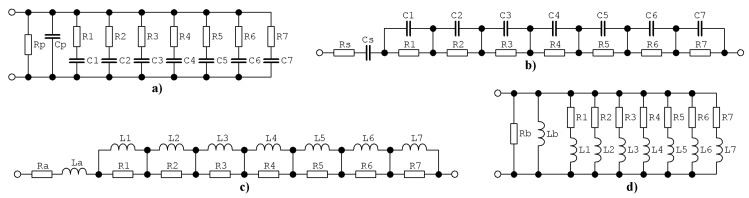
Basic network structures of fully passive ladder circuits dedicated for approximation of CPE: (**a**) series-parallel RC, (**b**) parallel-series RC, (**c**) parallel-series RL, (**d**) series-parallel RL.

CPEs can be successfully approximated in operational frequency range using various circuits; both passive and active. So far, the most common structure is the ladder network provided in Figure 1a. Input admittance of this fractal capacitor can be expressed as

(3) $$Y\left(s\right)=s\cdot C_{p}+\frac{1}{R_{p}}+\sum_{k=1}^{7}\frac{sC_{k}}{s\cdot C_{k}R_{k}+1}\;.$$

This function has 8 zeroes and 7 poles. Approximation itself begins with zero, i.e., a phase frequency response of the admittance is zero at DC, then begins to increase and finally, above approximated frequency band, asymptotically reaches 90°. A second simple passive ladder circuit dedicated for modeling two-terminal CPE with negative phase shift (fractal capacitor) is demonstrated in Figure 1b. In this case, input impedance is

(4) $$Y\left(s\right)=1/\left(R_{s}+\frac{1}{s\cdot C_{s}}+\sum_{k=1}^{7}\frac{R_{k}}{s\cdot C_{k}R_{k}+1}\right).$$

This function has 8 zeroes and the same number of poles. Approximation begins with zero located at zero frequency, i.e., phase shift of the admittance is 90° at DC, then starts to decrease to the desired value. The above upper frequency limit for approximation phase shift returns to zero and module of admittance is constant, as CPE behaves similar to a resistor. Fractal inductors can be implemented following the duality principle: resistors remain but value is inverted, capacitors are substituted by inductors, series connection of elements turns into parallel and vice versa. This approach, if applied on the schematic in Figure 1a, results into circuit provided in Figure 1c. Input impedance can be written in form

(5) $$Z\left(s\right)=R_{a}+s\cdot L_{a}+\sum_{k=1}^{7}\frac{s\cdot L_{ak}R_{ak}}{s\cdot L_{ak}+R_{ak}}\;.$$

Another promising structure of the fractal inductor is provided by means of Figure 1d where lossy inductors are employed. Admittance function of this two-terminal device is

(6) $$Z\left(s\right)=1/\left(\frac{1}{R_{b}}+\frac{1}{s\cdot L_{b}}+\sum_{k=1}^{7}\frac{1}{s\cdot L_{bk}+R_{bk}}\right)\;.$$

Equality (1) indicates how CPEs can be implemented using the active two-ports: as a cascade of eight bilinear sections or four biquadratic sections. Network function *F*(*s*) will be a voltage transfer, rather than immittance function. If suitable, two-port topology is adopted positions of individual zeroes and poles can be adjusted independently.

Design process toward fully passive ladder CPEs is thoroughly described in key papers [36,37]. However, frequencies of zeroes and poles, especially pairs located at the beginning of approximation, are too low to be implementable using common resistors and capacitors directly taken from standard fabrication series (E6, E12, E24, etc.). Series combination of resistors as well as parallel connection of capacitors do not solve this problem because a huge number of passive components are still required. However, large values of the capacitors can be created by using positive impedance converters and RL realizations are also up to date because we can take advantage of many known topologies of grounded and floating synthetic loss inductor. Thus, attention is paid only on the active realizations, both voltage-mode and current-mode, where realistic values of the circuit components can be found. Of course, a list of possible active realizations is by no way complete. Nevertheless, the proposed networks contain only cheap and off-the-shelf active elements.

## 3. Wideband CPE Dedicated for Lumped Chaotic Oscillators

Chaotic signals have several unique properties that predefined the utilization of chaotic oscillators in practical applications, such as long-time unpredictability of future states, absence of analytic solution in the closed form, extreme sensitivity to the changes of the initial conditions, continuous wideband frequency range, etc. Because of the latter case, CPEs applicable in the chaotic systems to model FO elements need to be wideband as well. Therefore, CPEs proposed in this section form alternative to audio CPEs are listed in paper [38], with larger phase error but wider bandwidth. Since exactly the same network structures for CPE approximation are proposed in both papers, it is possible to use the printed circuit boards depicted in [38]. Therein, to obtain the nearest numerical value required, each RC combination can be implemented by series and/or parallel interconnection of three resistors and three capacitors (fabricated in commercial series such as E6 or E12).

This section brings numerical values of the circuit components for different realizations of CPEs. Individual mathematical orders are provided as the sub-sections in ascending order; beginning with phase shift 9° (α = 1/10, behavior very close to resistor) and ending with 81° (α = 9/10, i.e., motion close to capacitor, inductor, ideal integrator, or differentiator). The total amount of 19 non-integer orders are chosen with respect to practical applications; each one represents a significant fraction between zero and one. Tabularized numerical values provided in each sub-section represent complete knowledge about behavior of developed wideband CPE in the form of RC passive-only ladder structure. Values provided for resistors and capacitors are calculated using algorithm described in fundamental papers [34,35] and rounded conveniently. Then, location of first and last zero-pole pair is slightly adjusted to enhance approximation bandwidth as much as possible. Concrete time constant of CPEs should be composed by series-parallel interconnection of real passive components taken from commercially available fabrication series with minimal tolerances (ideally 0.1% and/or 0.5% at maximum). Numerical values of CPEs are calculated so that the fundamental property of CPE, pseudo-capacitance or pseudo-inductance, is not considered for calculations and unified. This is, in fact, a value of module measured at angular frequency 1 rad/s, i.e., frequency 159 MHz Thus, it can be verified directly in the module frequency responses of the individual CPEs. For type I RC structure, module of CPE admittance is equal to 1/*R_P_* at DC frequency. For type II RC network, admittance of CPE is defined at very high frequencies and equals 1/*R_S_*.

### 3.1. Wideband CPE for Mathematical Order α = 1/10, Prescribed Phase Shift φ = ± 9°

This CPE has a start-up frequency *f*_0_ = 1/(2π*R*_0_*C*_0_) = 1/(2π·10^5^·10^−6^) = 1.6 Hz and utilize impedance constant 10^3^. Optimal values of resistors and capacitors associated with Figure 1a,b can be found within Table 1 and Table 2. Pseudo-capacitance is about 1.5 mF/s^9/10^ and 1.83 μF/s^9/10^ for Types I and II RC circuit, respectively.

### 3.2. Wideband CPE for Mathematical Order α = 1/9, Prescribed Phase Shift φ = ± 10°

This type of CPE has a start-up frequency *f*_0_ = 1/(2π*R*_0_*C*_0_) = 1/(2π·10^5^·10^−6^) = 1.6 Hz, uses impedance constant 10^4^ and optimal design values in the sense of Figure 1a,b can be found in Table 3 and Table 4. Pseudo-capacitance is close to value 143.6 μF/s^8/9^ for type I and 2 μF/s^8/9^ in the case of type II RC circuit, respectively.

### 3.3. Wideband CPE for Mathematical Order α = 1/5, Prescribed Phase Shift φ = ± 18°

This kind of CPE approximation has a start-up frequency *f*_0_ = 1/(2π*R*_0_*C*_0_) = 1/(2π·10^5^·10^−6^) = 1.6 Hz and utilize impedance constant 10^4^. Optimal values for design of this CPE can be found within Table 5 and Table 6. Estimated value of pseudo-capacitance is close to value 106.6 μF/s^4/5^ for type I and 2.76 μF/s^4/5^ for type II RC circuit, respectively.

### 3.4. Wideband CPE for Mathematical Order α = 2/9, Prescribed Phase Shift φ = ± 20°

This kind of approximation has start-up frequency *f*_0_ = 1/(2π*R*_0_*C*_0_) = 1/(2π·10^5^·10^−6^) = 1.6 Hz and by using impedance constant 10^4^. Optimal values for this CPE can be found in Table 7 and Table 8. Estimated pseudo-capacitance is close to 101.7 μF/s^7/9^ for type I and 2.76 μF/s^7/9^ for type II RC network, respectively.

### 3.5. Wideband CPE for Mathematical Order α = 1/4, Prescribed Phase Shift φ = ± 22.5°

This kind of approximation has start-up frequency *f*_0_ = 1/(2π*R*_0_*C*_0_) = 1/(2π·10^5^·10^−6^) = 1.6 Hz and uses impedance constant 10^4^. Optimal values for design of this CPE can be found inside Table 9 and Table 10. Estimated value of pseudo-capacitance is close to 96.8 μF/s^3/4^ for type I RC circuit and 2.8 μF/s^3/4^ for type II RC structure, respectively. This device is often called quarter capacitor or, in the case of two-port CPE, quarter integrator, respectively.

### 3.6. Wideband CPE for Mathematical Order α = 3/10, Prescribed Phase Shift φ = ± 27°

This CPE approximant has start-up frequency *f*_0_ = 1/(2π*R*_0_*C*_0_) = 1/(2π·10^5^·10^−6^) = 1.6 Hz, impedance constant is chosen to be 10^4^ and optimal values for design are provided within Table 11 and Table 12. Pseudo-capacitance value is approximately 94.2 μF/s^7/10^ for type I RC network and 2.84 μF/s^7/10^ for type II RC structure, respectively.

### 3.7. Wideband CPE for Mathematical Order α = 1/3, Prescribed Phase Shift φ = ± 30°

This kind of approximation has start-up frequency *f*_0_ = 1/(2π*R*_0_*C*_0_) = 1/(2π·10^5^·10^−6^) = 1.6 Hz and uses impedance constant 10^4^. Optimal values for design of this CPE can be found in Table 13 and Table 14. Estimated value of pseudo-capacitance is about 92.27 μF/s^2/3^ for type I CPE and 2.8 μF/s^2/3^ for type II CPE, respectively.

### 3.8. Wideband CPE for Mathematical Order α = 2/5, Prescribed Phase Shift φ = ± 36°

This CPE approximation has a start-up frequency *f*_0_ = 1/(2π*R*_0_*C*_0_) = 1/(2π·10^5^·10^−6^) = 1.6 Hz and utilize impedance constant 10^4^. Optimal values for design of this CPE can be found in Table 15 and Table 16. Estimated pseudo-capacitance is close to 87.5 μF/s^3/5^ for type I and 2.24 μF/s^3/5^ for type II RC network, respectively.

### 3.9. Wideband CPE for Mathematical Order α = 4/9, Prescribed Phase Shift φ = ± 40°

This kind of approximation has a start-up frequency *f*_0_ = 1/(2π*R*_0_*C*_0_) = 1/(2π·0.8·10^5^·10^−6^) = 2 Hz and uses high impedance constant 10^4^. Optimal values for immediate design of this CPE can be found inside Table 17 and Table 18. Estimated value of pseudo-capacitance is 83.77 μF/s^5/9^ for type I and 2.9 μF/s^5/9^ for type II RC circuit, respectively.

### 3.10. Wideband CPE for Mathematical Order α = 1/2, Prescribed Phase Shift φ = ± 45°

This kind of CPE approximation has a start-up frequency *f*_0_ = 1/(2π*R*_0_*C*_0_) = 1/(2π·0.8·10^5^·10^−6^) = 2 Hz and utilize high impedance constant 10^4^. Optimal numerical values for design of this CPE can be found in Table 19 and Table 20. Estimated value of pseudo-capacitance is close to 83.4 μF/s^1/2^ for type I and 2.533 μF/s^1/2^ for type II RC circuit, respectively. This device is known as a half capacitor, respectively.

### 3.11. Wideband CPE for Mathematical Order α = 5/9, Prescribed Phase Shift φ = ± 50°

This kind of approximation has start-up frequency *f*_0_ = 1/(2π*R*_0_*C*_0_) = 1/(2π·0.8·10^5^·10^−6^) = 2 Hz and by using a high impedance constant 10^4^. Optimal values for design of this CPE can be found in Table 21 and Table 22. Estimated value of pseudo-capacitance is close to 84 μF/s^4/9^ (type I) and 2.2 μF/s^4/9^ (type II), respectively.

### 3.12. Wideband CPE for Mathematical Order α = 3/5, Prescribed Phase Shift φ = ±54°

This approximation begins with a frequency *f*_0_ = 1/(2π*R*_0_*C*_0_) = 1/(2π·0.6·10^5^·10^−6^) = 2.65 Hz and utilize impedance constant 10^4^. Optimal values for immediate design of this CPE can be found in Table 23 and Table 24. Roughly estimated value of pseudo-capacitance is close to 83.3 μF/s^2/5^ for type I and 2.2 μF/s^2/5^ for type II RC network, respectively.

### 3.13. Wideband CPE for Mathematical Order α = 2/3, Prescribed Phase Shift φ = ± 60°

This approximation begins with frequency *f*_0_ = *1*/(2π*R*_0_*C*_0_) = 1/(2π·6·10^5^·10^−7^) = 2.65 Hz and utilize impedance constant 10^6^. Optimal values for complete design of this CPE can be found in Table 25 and Table 26. Estimated value of pseudo-capacitance is close to 863.8 nF/s^1/3^ in the case of type I RC network and 157.6 nF/s^1/3^ for type II RC circuit, respectively.

### 3.14. Wideband CPE for Mathematical Order α = 7/10, Prescribed Phase Shift φ = ± 63°

Begins with frequency *f*_0_ = 1/(2π*R*_0_*C*_0_) = 1/(2π·5·10^5^·10^−7^) = 3.2 Hz and utilize impedance constant 10^6^. Optimal values for design of this CPE can be found in Table 27 and Table 28. Pseudo-capacitance is near to value 887 nF/s^3/10^ for type I RC ladder network and 139.5 nF/s^3/10^ in the case of type II RC passive ladder approximant, respectively.

### 3.15. Wideband CPE for Mathematical Order α = 3/4, Prescribed Phase Shift φ = ± 67.5°

Begins with frequency *f*_0_ = *1*/(2π*R*_0_*C*_0_) = 1/(2π·5·10^5^·10^−7^) = 3.2 Hz and utilize impedance constant 10^6^. Optimal values of passive off-the-shelf components to design this CPE can be found in Table 29 and Table 30. Pseudo-capacitance is about 944 nF/s^1/4^ for type I and 104.5 nF/s^1/4^ for type II RC ladder circuit, respectively.

### 3.16. Wideband CPE for Mathematical Order α = 7/9, Prescribed Phase Shift φ = ± 70°

Approximation of CPE begins at frequency *f*_0_ = 1/(2π*R*_0_*C*_0_) = 1/(2π·3·10^5^·2·10^−7^) = 2.65 Hz, impedance constant is chosen to be 10^6^. Optimal numerical values of circuit components can be found in Table 31 and Table 32. Pseudo-capacitance is roughly estimated to be 1 φF/s^2/9^ for type I and 174.2 nF/s^2/9^ for type II RC circuit, respectively.

### 3.17. Wideband CPE for Mathematical Order α = 4/5, Prescribed Phase Shift φ = ± 72°

Fundamental frequency of this CPE is *f*_0_ = 1/(2π*R*_0_*C*_0_) = 1/(2π·5·10^5^·10^−7^) = 3.2 Hz, impedance constant was set to 10^6^, and values of passive circuit elements are provided in Table 33 and Table 34. Estimated value of pseudo-capacitance is close to value 1.06 μF/s^1/5^ in the case of type I and 74 nF/s^1/5^ for type II RC ladder network, respectively.·

### 3.18. Wideband CPE for Mathematical Order α = 8/9, Prescribed Phase Shift φ = ± 80°

Start-up frequency is *f*_0_ = *1*/(2π*R*_0_*C*_0_) = 1/(2π·2·10^5^·2·10^−7^) = 4 Hz, high impedance constant set to 10^7^. Optimal values can be found within Table 35 and Table 36. Pseudo-capacitance is close to 143.4 nF/s^1/9^ (type I) and 69.32 nF/s^1/9^ (type II), respectively.

### 3.19. Wideband CPE for Mathematical Order α = 9/10, Prescribed Phase Shift φ = ± 81°

Start-up frequency is *f*_0_ = *1*/(2π*R*_0_*C*_0_) = 1/(2π·1.8·10^5^·2·10^−7^) = 4.4 Hz, high impedance constant is 2·10^7^. Optimal values can be found in Table 37 and Table 38. Pseudo-capacitance is close to 76.5 nF/s^1/10^ (type I) and 58.65 nF/s^1/10^ (type II), respectively.

### 3.20. Numerical Verification of Wideband CPEs

**Figure 2:**
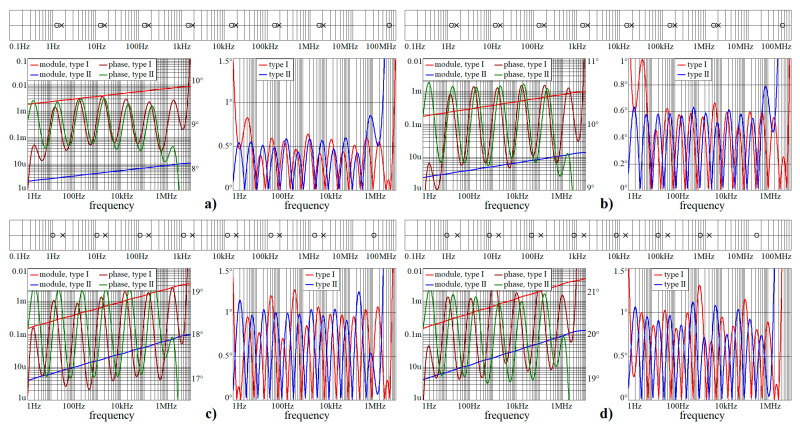
Numerical verification of designed wideband CPEs for all orders considered in this paper. Locations of zeroes and poles on frequency axis of CPE considered as admittance two-terminal device, module (red and blue) and phase (brown and green) frequency response, absolute error of first (red) and second (blue) type of RC approximation circuit: (**a**) α = 1/10, (**b**) α = 1/9, (**c**) α = 1/5, and (**d**) α = 2/9.

**Figure 3:**
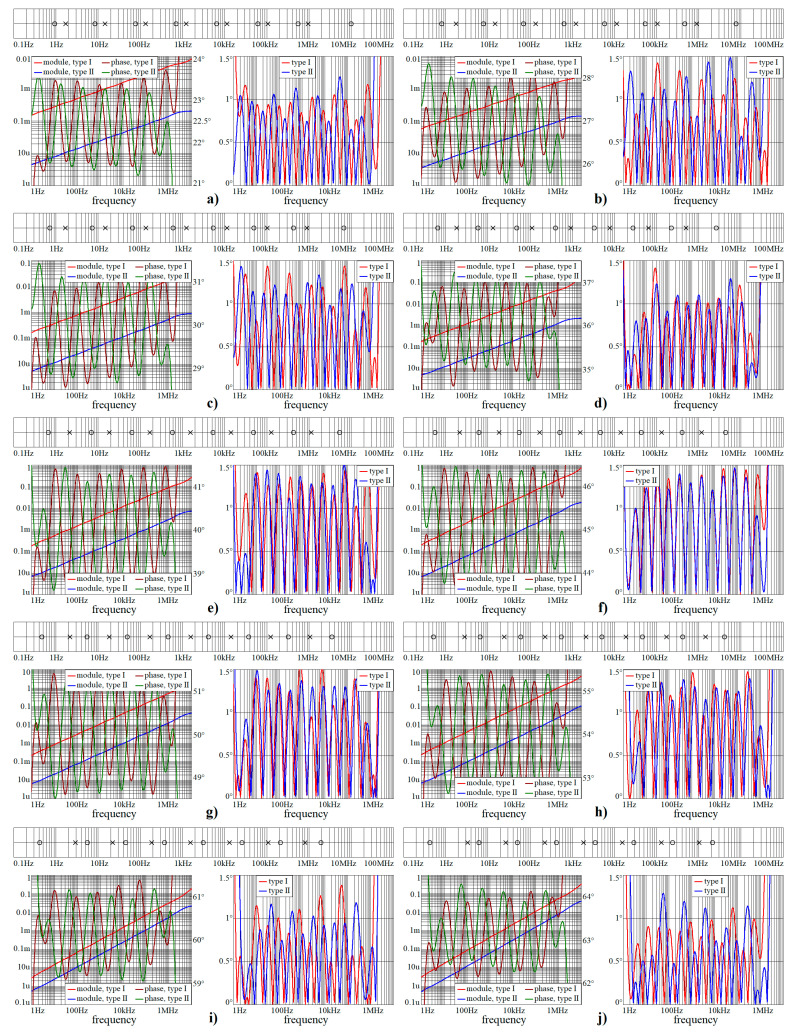
Numerical verification of designed wideband CPEs for all orders considered in this paper, continuation of the previous figure: (**a**) α = 1/4, (**b**) α = 3/10, (**c**) α = 1/3, (**d**) α = 2/5, (**e**) α = 4/9, (**f**) α = 1/2, (**g**) α = 5/9, (**h**) α = 3/5, (**i**) α = 2/3, and (**j**) α = 7/10.

**Figure 4:**
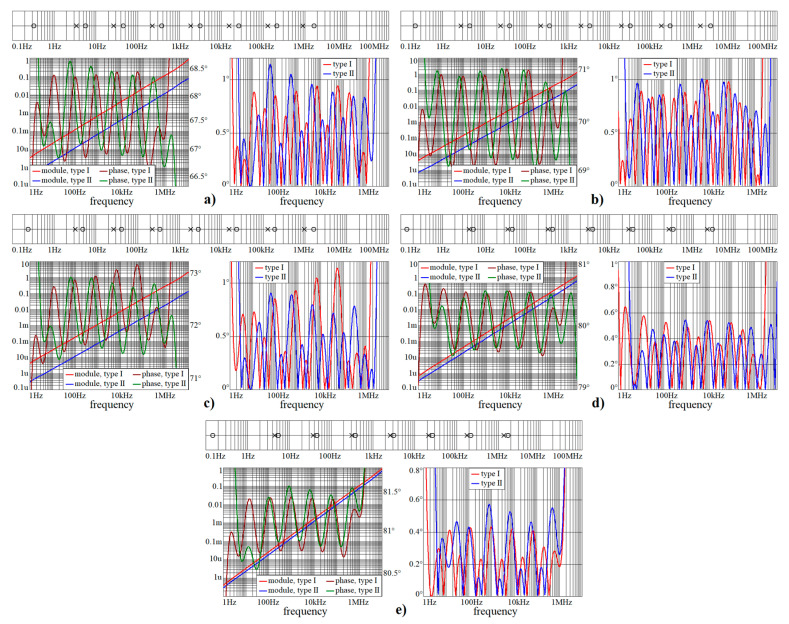
Numerical verification of designed wideband CPEs for all orders considered in this paper, continuation of the previous figure: (**a**) α = 3/4, (**b**) α = 7/9, (**c**) α = 4/5, (**d**) α = 8/9, and (**e**) α = 9/10.

This sub-section shows numeric verification of wideband CPEs in Mathcad. Obtained results are provided via Figure 2, Figure 3 and Figure 4. Both frequency responses, i.e., module and phase, and absolute errors of first and second RC ladder structure, are calculated in frequency range starting with 1 Hz and ending at 10 MHz. As required, phase error is below 1.5°. Within these pictures, the locations of zeroes and poles of complex admittance function are also provided, from 100 mHz up to 100 MHz.

**Figure 5:**
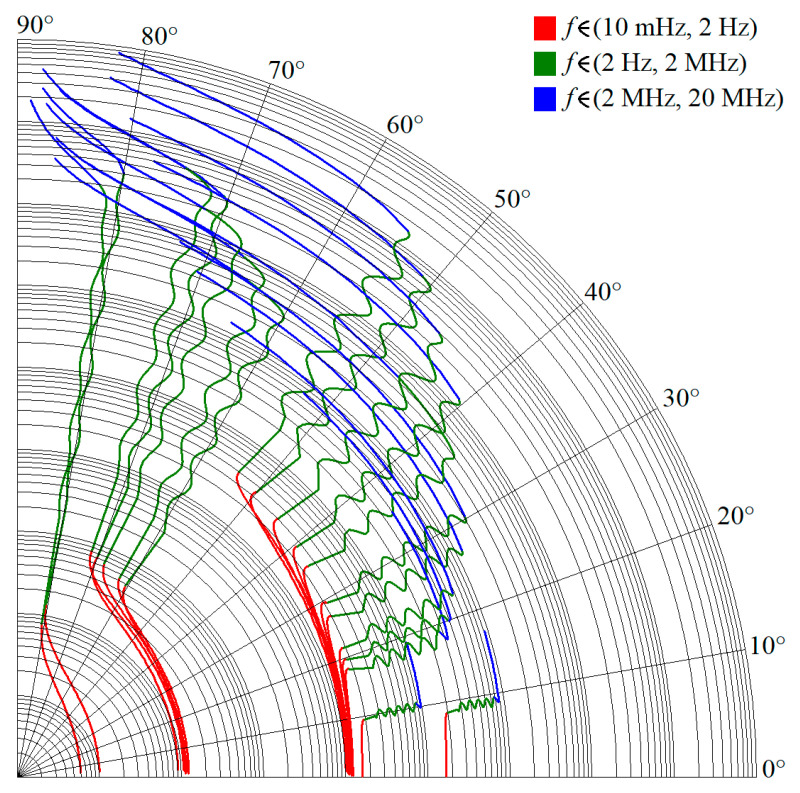
Polar plots of complex frequency responses of designed CPEs; series-parallel RC structures.

**Figure 6:**
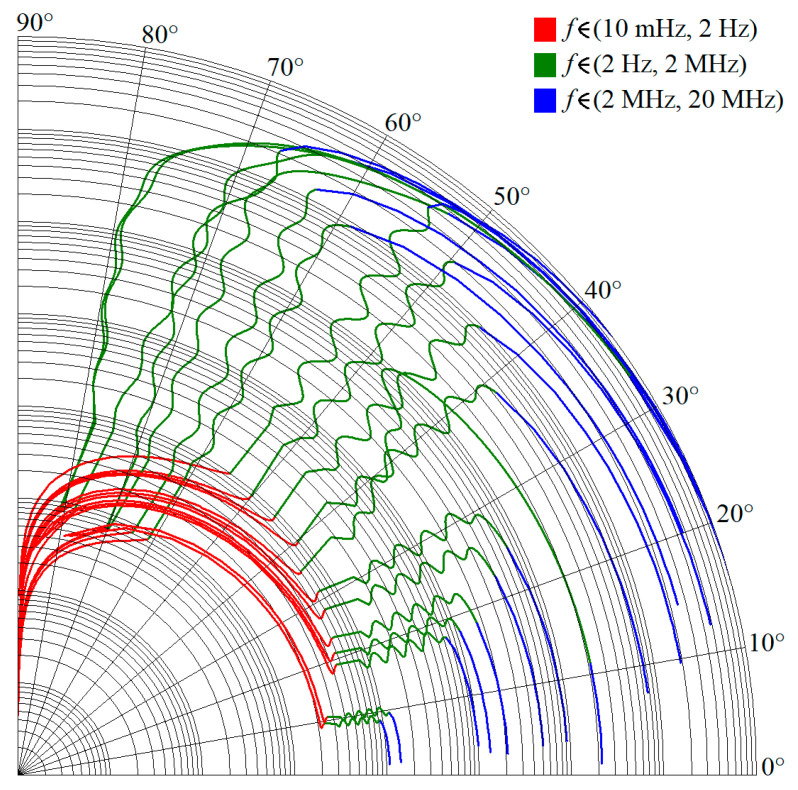
Polar plots of complex frequency responses of designed CPEs; parallel-series RC structures.

Note that phase error is always smaller than ±1.5° in the required frequency band from 3 Hz up to 1 MHz, i.e., phase frequency response is located within predefined tolerance channel. Figure 5 shows polar plots of complex admittance functions for individual RC configurations in the sense of Figure 1a. Figure 6 demonstrate the same for passive CPE approximants given in Figure 1b.

## 4. Transformations Associated with Passive CPEs

As previously mentioned, the CPEs suggested in the previous section are designed for the frequency band from 3 Hz up to 1 MHz, i.e., in nearly six decades. Additionally, impedance constant of individual approximation circuits is different, so that numerical values of resistors and capacitors are reasonable. This is good for chaotic systems if time constant is chosen properly, as demonstrated in upcoming section of this paper. However, another application may require approximation of CPEs valid in different frequency bands, for example, subsonic or ultrasound bands. In such case, frequency normalization is able to shift whole phase frequency response down or up along the frequency axis without changes of its shape (e.g., phase ripple does not become deformed). Doing so, module frequency response does not change. By introducing the impedance norm, we can shift module frequency response vertically down or up while phase frequency response remains exactly the same. This allows us to recalculate all approximation of CPEs to have a pseudo-capacitance equal to one F/s^1−α^.

Horizontal movement of phase frequency response to the left (right) proportional to size Ω < 1 (Ω > 1) can be done by dividing all capacitors by Ω, resistors stand unchanged. Vertical movement of module frequency response down (up) proportional to value ξ can be done by dividing all capacitors by ξ while all resistors are multiplied by value ξ. In practice, both transformations are performed simultaneously. This operation can be expressed as

(7) $$C_{k}^{new}=C_{k}^{old}/\left(\Omega \cdot \xi \right)\;\;\;\;\;\;\;\;\;R_{k}^{new}=R_{k}^{old}\cdot \xi ,$$

where *k* is index of circuit component including those elements denoted as *C_p_*, *R_p_*, *C_s_*, and *R_s_*. Both transformations mentioned above renders CPEs designed in this paper more flexible, universal, and customizable for concrete practical application. It is also not restricted for passive ladder networks proposed here; both transformations can be directly used for any RC structure, i.e., also for audio CPEs designed in paper [38], RC tree networks, active RC topologies (only frequency norms work in general), etc. For design of FO chaotic oscillators, value Ω should be chosen carefully so that the natural harmonic component of the chaotic signal is in the middle of frequency range (in geometrical sense) where CPE approximation is valid. Roughly speaking, the entire frequency spectrum of chaotic signal should be covered by CPE approximation. This proposition holds in general: frequency band of processed signals should be covered by frequency range of CPE approximation.

## 5. Wideband CPE as Part of Chaotic System

It is well known that the dynamical behavior that is both bounded and extremely sensitive to tiny deviations of initial conditions can be generated by third-order autonomous deterministic dynamical system with at least one scalar nonlinearity. Besides initial conditions, behavior of both autonomous and driven chaotic systems is sensitive to the internal parameters as well. Small deviations can cause deformation and collapse of dense strange attractor predefined by numerical integration. Therefore, the design of FO chaotic oscillator requires very good approximation of CPE over wide frequency range. Practical experience with approximated CPEs confirms that all mathematical orders are very sensitive to numerical values of resistors and capacitors. Thus, general recommendation during construction is to make a careful selection and the measure real value of all passive component before assembly to PCB.

**Figure 7:**
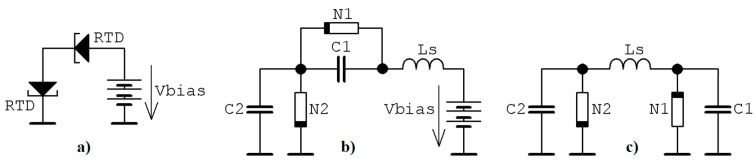
Different structures of the analyzed memory: (**a**) principal concept, (**b**) high frequency model, (**c**) electronic circuit after transformation of AVC of both RTDs toward origin.

Quite recently, it has been proved that robust chaotic waveforms can be generated by binary memory composed by two coupled resonant tunneling diodes (RTD) [39] approximated by either piecewise linear (PWL) [40] or cubic polynomial function [41]. Both diodes possess typical N-type ampere-voltage characteristics (AVC) and three degrees of freedom required for chaos evolution are obtained due to the parasitic features of RTDs observed on the high frequencies. These can be modeled by a pair of junction capacitances and lead inductance [42]. Basic structure of static ternary memory cell is provided by means of Figure 7a. Two RTDs are connected in series together with biasing voltage responsible for proper geometrical configuration of vector field. In this operational condition either robustness of three stable states or potential stability problem is achieved. If high-frequency models of RTDs are considered, simple circuitry given in Figure 7b can be derived. Without loss of generality PWL AV curves of both RTDs can be shifted toward origin so that biasing voltage source can be removed. After small rearrangement of network components simple circuitry given in Figure 7c can be obtained. Behavior of resulting dynamical system can be described by a following set of first-order ordinary differential equations

(8) $$C_{1}\frac{d}{dt}v_{1}=-i_{L}+f_{1}\left(v_{1}\right)\;\;\;\;\;\;\;\;\;\;C_{2}\frac{d}{dt}v_{2}=i_{L}-f_{2}\left(v_{2}\right)\;\;\;\;\;\;\;\;\;\;L\frac{d}{dt}i_{L}=v_{1}-v_{2},$$

where *f*_1_ and *f*_2_ are scalar three-segment odd-symmetrical saturation-type PWL functions. Individual *k*-th PWL function can be expressed as

(9) $$\begin{array}{c} \left|v_{k}\right|\leq \beta_{k}\;\to f_{k}=g_{inner}^{k}\cdot v_{k}\\ v_{k}>\beta_{k}\;\to f_{k}=g_{outer}^{k}\left(v_{k}-\beta_{k}\right)+g_{inner}^{k}\cdot \beta_{k}\\ v_{k}<-\beta_{k}\;\to f_{k}=g_{outer}^{k}\left(v_{k}+\beta_{k}\right)-g_{inner}^{k}\cdot \beta_{k}, \end{array}$$

where *g^k^_inner_* and *g^k^_outer_* is slope of *k*-th PWL function in inner and outer segments respectively and *β_k_* stands for breakpoint voltage. Locations of fixed points can be determined via two voltages

(10) $$v_{x}=\frac{\beta_{1}\left(g_{outer}^{1}-g_{inner}^{1}\right)+\beta_{2}\left(g_{inner}^{2}-g_{outer}^{2}\right)}{g_{outer}^{1}-g_{outer}^{2}}\;\;\;\;\;\;\;\;\;\;v_{y}=\frac{\beta_{1}\left(g_{outer}^{1}-g_{inner}^{1}\right)}{g_{inner}^{2}-g_{outer}^{1}}.$$

Using these auxiliary numbers, positions of the equilibrium points (if exist) are **x**_e1_ = [*v_x_*, *v_x_*, *f*_2_(*v_x_*)]^T^, **x**_e2_ = [*v_y_*, *v_y_*, *f*_2_(*v_y_*)]^T^, **x**_e3_ = [0, 0, 0]^T^, **x**_e4_ = [−*v_x_*, −*v_x_*, *f*_2_(−*v_x_*)]^T^, and **x**_e5_ = [−*v_y_*, −*v_y_*, *f*_2_(−*v_y_*)]^T^. In each segment of vector field, local behavior is uniquely determined by eigenvalues, i.e., roots of characteristic polynomial

(11) $$s^{3}+\frac{L\left(C_{1}\cdot g^{2}-C_{2}\cdot g^{1}\right)}{C_{1}\cdot C_{2}\cdot L}s^{2}+\frac{C_{1}+C_{2}-L\cdot g^{1}g^{2}}{C_{1}\cdot C_{2}\cdot L}s+\frac{g^{2}-g^{1}}{C_{1}\cdot C_{2}\cdot L}=0,$$

where *g^n^* is slope of *n*-th PWL function in the investigated segment of vector field.

**Figure 8:**
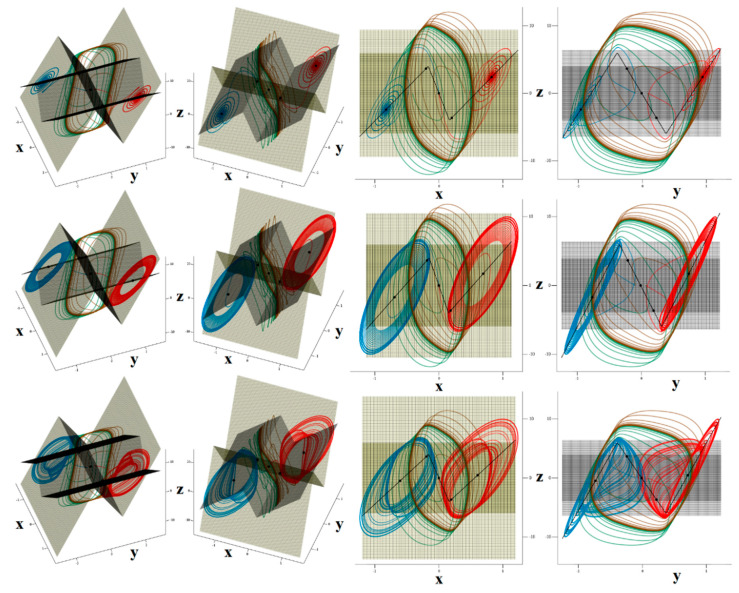
Localization of attractors, fixed points (black dots) and PWL functions for memory having different values of transconductance slope *g*^2^_outer_: *g*^2^_outer_ = 14 S (upper row), *g*^2^_outer_ = 15 S (middle row) and *g*^2^_outer_ = 18 S (lower row), initial conditions are set to points: **x**_0_ = (0, 0.1, 0)^T^ (red), **x**_0_ = (0, −0.1, 0)^T^ (blue), **x**_0_ = (−0.5, 0.1, 0)^T^ (green) and **x**_0_ = (0.5, −0.1, 0)^T^ (brown).

**Figure 9:**
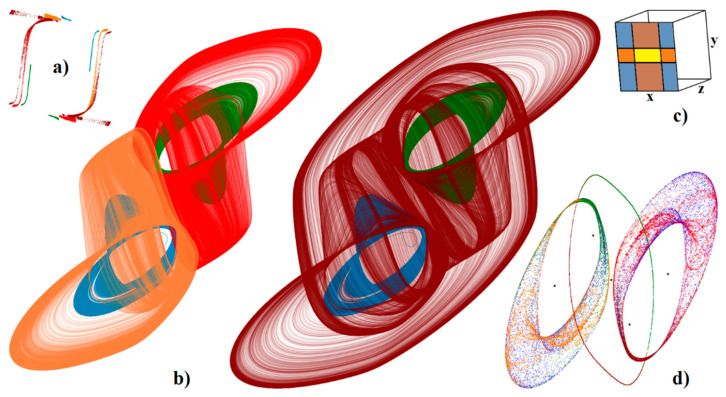
3D visualization of the mutual geometrical relations between calculated chaotic attractors: mirrored single-spirals (blue and green), mirrored funnels (orange and red), double-scroll (brown). Individual plots: (**a**) Poincaré section defined by plane *z* = 0, (**b**) perspective views on strange attractors, (**c**) state space rotation used for the best visualization of presented strange attractors and its separation into segments, (**d**) sensitivity of both single-scroll attractors to tiny changes of the initial conditions—black dots represent fixed points. See text for further clarification.

Several methods of how to distinguish between regular and irregular behavior of arbitrary order mathematical model have been developed and published. Some of them are based on calculation of flow quantifier such as the largest Lyapunov exponent (LLE), metric dimensions, or by using return maps. Interesting reading about this topic is provided in paper [43] and references are cited therein. Utilization of such an algorithm as an objective function for optimization leads to set of normalized values that causes memory to behave chaotically, namely *c*_1_ = 10 F, *c*_2_ = 6 F, *l* = 100 mH, *g*^1^_inner_ = −20 S, *g*^1^_outer_ = 8 S, *β*_1_ = 200 mV, *g*^2^_inner_ = −15 S, *g*^2^_outer_ = 18 S, and *β*_2_ = 400 mV. All state trajectories plotted in this section were numerically integrated using Mathcad 15 and build-in fourth order Runge-Kutta method having fixed step size. The type of the dynamical behavior of the memory strongly depends on the shapes of both PWL functions. For example, numerically observed attractors for different slope of outer segments associated with second RTD are demonstrated in Figure 8. The first two columns provide a 3D perspective view on state space while the third and fourth column are two Monge projections of the same situation. Note that the well-known single-scroll strange attractor is obtained for value *g*^2^_outer_ = 18 S. Here, final time for numerical integration was set to 200 and time step 0.01. Further experimentations reveal that funnel and double-scroll chaotic attractor can be also robust solution of analyzed set of differential equations, namely for normalized values *c*_2_ = 4.5 F, *l* = 150 mH and *c*_2_ = 6 F, *l* = 170 mH, *g*^2^_outer_ = 20 S, respectively. Remaining internal parameters of memory system are unchanged. Position of these attractors within state space is visualized by means of Figure 9. Final time was set to 10^4^, time step 0.1 and initial conditions were **x**_0_ = (0, 0, ±0.1)^T^ for single-spirals and **x**_0_ = (0, 0, ±0.1)^T^ for funnels. Due to vector field symmetry, two lateral strange attractors can merge, forming large attracting set that enters all state space segments.

A key feature of chaos is the extreme sensitivity of the system behavior to the tiny changes of initial conditions. This unique property is proved in Figure 9d, where five groups of 10^4^ initial conditions were integrated with a final time of 100 and time step of 0.1 (ending state is plotted). Each group is generated in the close neighborhood of some fixed point (black dots) distinguished by colors (**x**_e1_ red, **x**_e2_ blue, **x**_e3_ green, **x**_e4_ orange, and **x**_e5_ brown) using normal distribution with mean deviation 10^−3^. Note that self-excitation process of the limit cycle and both mirrored single-spiral attractors is verified.

Let’s see what kind of vector field geometry forms double-scroll attractor newly presented in this paper. This attractor occupies all affine segments of the state space, i.e., dynamics of memory is uniquely determined by eigenvalues and eigenspaces associated with all fixed points. For numerical set of parameters given above, formula (11) returns the following results: saddle-focus with unstable eigenplane in blue segments in the sense of Figure 9c, a full saddle focus repellor with spiral movement in orange areas, stable spiral combined with stable vector movement in brown regions, and finally a saddle node with stability index one within the yellow region.

**Figure 10:**
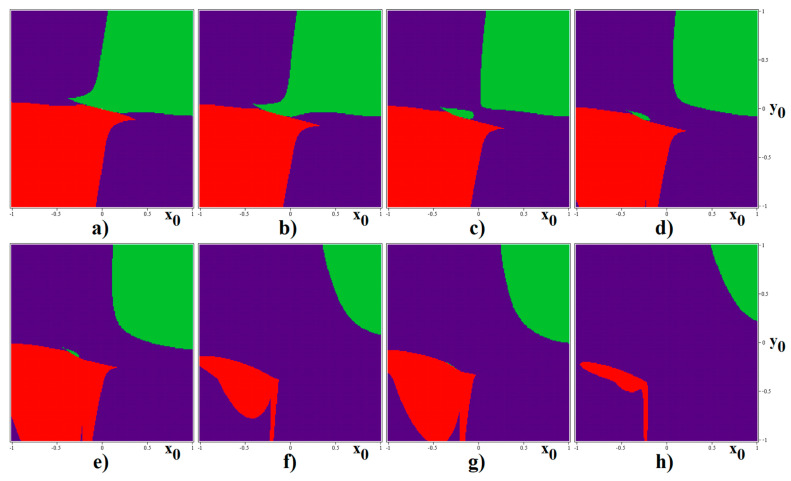
BA of analyzed memory with basic set of parameters (*g*^2^_outer_ = 18 S) leading to the separated single-spiral attractors, sandwiched horizontal slices of state space defined by the following planes: (**a**) *z*_0_ = 0, (**b**) *z*_0_ = 1, (**c**) *z*_0_ = 1.5, (**d**) *z*_0_ = 2, (**e**) *z*_0_ = 2.5, (**f**) *z*_0_ = 4, (**g**) *z*_0_ = 5, and (**h**) *z*_0_ = 6.

**Figure 11:**
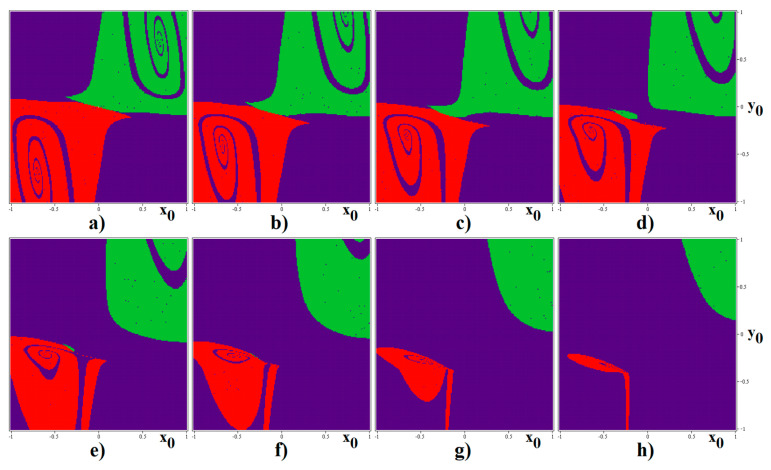
BA of analyzed memory with basic set of parameters (*g*^2^_outer_ = 20 S) leading to the separated single-spiral attractors, horizontal slices of state space defined by the planes: (**a**) *z*_0_ = 0, (**b**) *z*_0_ = 1, (**c**) *z*_0_ = 1.5, (**d**) *z*_0_ = 2, (**e**) *z*_0_ = 3, (**f**) *z*_0_ = 4, (**g**) *z*_0_ = 5, and (**h**) *z*_0_ = 6.

As nicely demonstrated by the chaotic Chua´s oscillator [44] or memory cell [45], similar to that analyzed in this work, calculation of basins of attraction (BA) for different limit sets can lead to the interesting, unexpected results. For two values of transconductance slopes *g*^2^_outer_, namely 18 S and 20 S, graphical visualization of BA is provided by means of Figure 10 and Figure 11 respectively. In these graphs, the blue color represents the limit cycle, yellow is the fixed-point equilibrium, and red and green marks left and right chaotic attractor. Due to computational time demands, a relatively small state space cube with size 2 × 2 × 4,5 was investigated; with step size of the initial conditions 0.01 × 0.01 × 0.5. Due to vector field symmetry caused by PWL functions, BA are also symmetrical with respect to *x* = 0, *y* = 0, and *z* = 0 axis. Note that, in the case of *g*^2^_outer_ = 18 S, geometrical structures of individual BA seem to be quite simple. On the other hand, transconductance slope equal to *g*^2^_outer_ = 20 S leads to a much more complicated snake-like regions ending into periodic solution. It should be noted that the chaotic attractors discovered in this paper are to self-excited. However, the existence of the hidden chaotic attractors is not excluded since, in the sense of initial conditions, investigated space is too small and grid large. Remember that, even in the case of the “old” and well-known Chua´s oscillator, which was analyzed more than three decades, hidden strange attractors were discovered quite recently [46].

**Figure 12:**
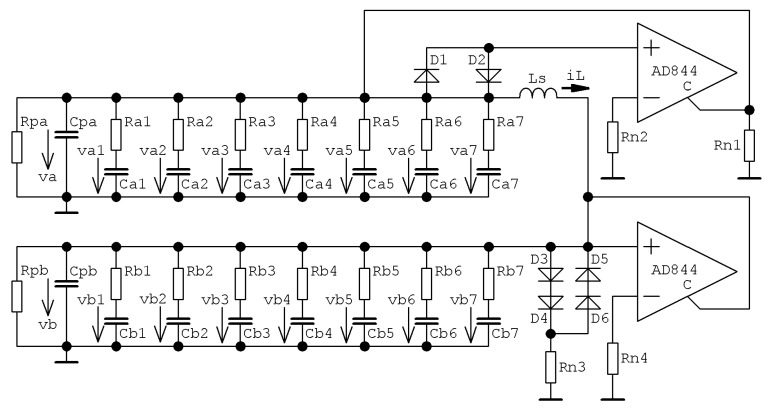
Complete analog circuitry realization of ternary memory with real CPEs approximated by passive RC network in function as FO capacitors, total mathematical order of this circuit is 17.

By introducing FO derivatives to differential equations that describe voltage vs. current flowing through capacitors, we get

(12) $$Y_{1}\frac{d^{\alpha }}{dt^{\alpha }}v_{1}=-i_{L}+f_{1}\left(v_{1}\right)\;\;\;\;\;\;\;\;\;\;Y_{2}\frac{d^{\beta }}{dt^{\beta }}v_{2}=i_{L}-f_{2}\left(v_{2}\right)\;\;\;\;\;\;\;\;\;\;L\frac{d}{dt}i_{L}=v_{1}-v_{2},$$

where *Y*_1,2_ is the pseudo-capacitance of first and second FO capacitor, respectively. If the capacitor is replaced by the approximation circuit depicted in Figure 1a, current vs. voltage relation changes into

(13) $$i=C_{1}\frac{d}{dt}v\;\;\;\;\;\to \;\;\;\;\;i=C_{p}\frac{d}{dt}v+\frac{v}{R_{p}}+\sum_{k=1}^{7}\frac{v-v_{k}}{R_{k}}\;\;\;\;\;\;\;\;\;\;\frac{d}{dt}v_{k}=\frac{1}{C_{k}R_{k}}\left(v-v_{k}\right),$$

where *v* and *i* is external voltage and current across CPE and *v_k_* are the internal nodes of CPE practically invisible to the rest of circuit. Note that state vector associated with memory changes from basic set **x** = (*v*_1_, *v*_2_, *i_L_*)^T^ into more complex form **x** = (*v_a_*, *v*_b_, *i_L_*, *v_a_*_1_, *v_a_*_2_, *v_a_*_3_, *v_a_*_4_, *v_a_*_5_, *v_a_*_6_, *v_a_*_7_, *v_b_*_1_, *v_b_*_2_, *v_b_*_3_, *v_b_*_4_, *v_b_*_5_, *v_b_*_6_, *v_b_*_7_)^T^. It means that each FO capacitor increases in order of final mathematical model by number equivalent to order of CPE approximation. For definition of individual state variables and complete schematic of FO memory, see Figure 12. Mathematical model of this circuitry can be expressed as *d***x**/*dt* = **A**·**x** + **f**(**x**), where entries of state matrix **A**^2*n*+3^, *n* = 7 is order of CPE approximation, are

(14) $$\begin{array}{c} A_{1,k}=\frac{-1}{C_{pa}}\left(\frac{1}{R_{pa}}+\sum_{k=1}^{7}\frac{1}{R_{ak}}\right)\;\;\;\;\;A_{2,k}=\frac{-1}{C_{pb}}\left(\frac{1}{R_{pb}}+\sum_{k=1}^{7}\frac{1}{R_{bk}}\right)\;\;\;\;\;A_{1,3}=\frac{1}{C_{pa}}\;\;\;\;\;A_{2,3}=\frac{1}{C_{pb}}\\ A_{3,1}=-A_{3,2}=\frac{1}{L}\;\;\;\;\;{A_{k,1}|}_{k=4,\;\ldots ,\;10}=\frac{1}{C_{a\left(k-3\right)}R_{a\left(k-3\right)}}\;\;\;\;\;{A_{1,k}|}_{k=4,\;\ldots ,\;10}=\frac{1}{C_{pa}R_{a\left(k-3\right)}}\\ {A_{k,2}|}_{k=11,\;\ldots ,\;17}=\frac{1}{C_{b\left(k-10\right)}R_{b\left(k-10\right)}}\;\;\;\;\;{A_{k,k}|}_{k=4,\;\ldots ,\;10}=\frac{-1}{C_{a\left(k-3\right)}R_{a\left(k-3\right)}}\;\\ {A_{k,k}|}_{k=11,\;\ldots ,\;17}=\frac{1}{C_{b\left(k-10\right)}R_{b\left(k-10\right)}}\;\;\;\;\;{A_{2,k}|}_{k=1,\;\ldots ,\;7}=\frac{1}{C_{pa}R_{ak}}\;\;\;\;\;A_{3,1}=-A_{3,2}=\frac{1}{L}, \end{array}$$

where the components of column vector **f** are *f*_1_(*v*_1_) and *f*_2_(*v*_2_) given by PWL function (9). Numerical values of components *R_pa_*, *C_pa_*, *R_pb_*, *C_pb_*, *R_ak_*, *C_ak_*, *R_bk_*, *C_bk_* for *k* = 1, 2, …, 7 can be adopted directly from Section 3 of this paper. The chaotic oscillator is designed so that only off-the-shelf electronic components are required. Used diodes are BAT 63 because of the low forward voltage of about 200 mV. Buffered voltage output of the integrated circuit AD844 can be utilized to trace voltages across FO capacitors. The whole network is fed by using symmetrical ±15 V voltage supply. Note that only integer-order nature of memory´s lead inductance is assumed.

**Figure 13:**
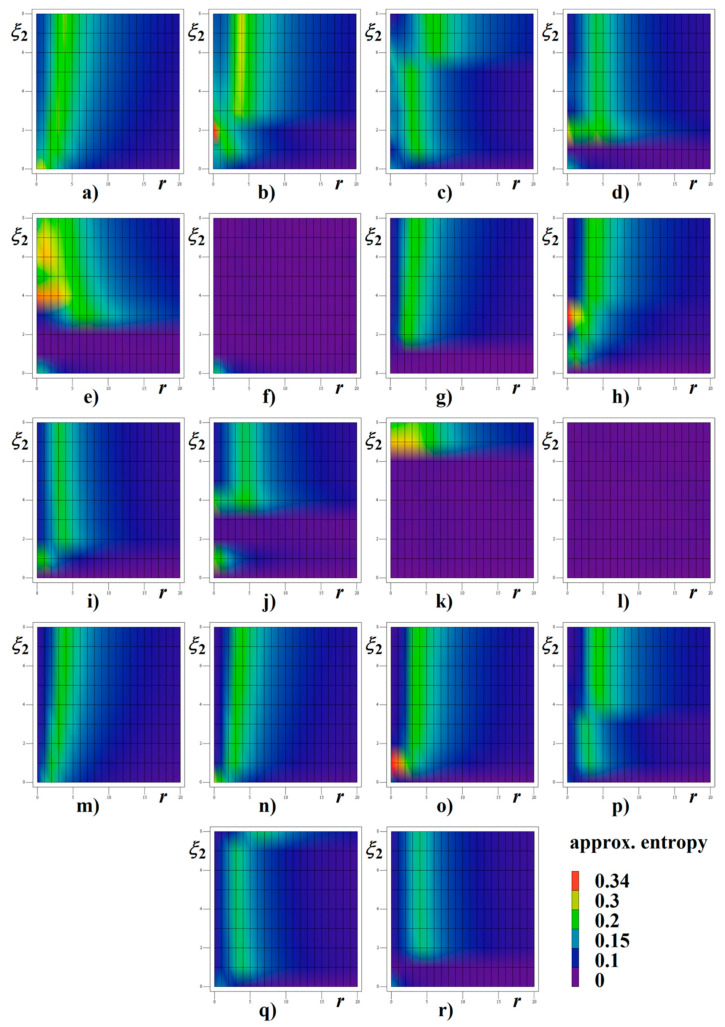
Approximate entropy calculated for memory with two CPEs of different orders *α* and *β*, associated impedance norms *ξ*_1_, *ξ*_2_ and threshold *r*: (**a**) *α* = *β* = 9/10, *ξ*_1_ = 1, (**b**) *α* = *β* = 9/10, *ξ*_1_ = 2, (**c**) *α* = *β* = 9/10, *ξ*_1_ = 3, (**d**) *α* = *β* = 9/10, *ξ*_1_ = 4, (**e**) *α* = *β* = 9/10, *ξ*_1_ = 5, (**f**) *α* = *β* = 9/10, *ξ*_1_ = 6, (**g**) *α* = 9/10, *β* = 8/9, *ξ*_1_ = 1, (**h**) *α* = 9/10, *β* = 8/9, *ξ*_1_ = 2, (**i**) *α* = 9/10, *β* = 8/9, *ξ*_1_ = 3, (**j**) *α* = 9/10, *β* = 8/9, *ξ*_1_ = 4, (**k**) *α* = 9/10, *β* = 8/9, *ξ*_1_ = 5, (**l**) *α* = 9/10, *β* = 8/9, *ξ*_1_ = 6, (**m**) *α* = 8/9, *β* = 9/10, *ξ*_1_ = 1, (**n**) *α* = 8/9, *β* = 9/10, *ξ*_1_ = 2, (**o**) *α* = 8/9, *β* = 9/10, *ξ*_1_ = 3, (**p**) *α* = 8/9, *β* = 9/10, *ξ*_1_ = 4, (**q**) *α* = 8/9, *β* = 9/10, *ξ*_1_ = 5, (**r**) *α* = 8/9, *β* = 9/10, *ξ*_1_ = 6.

**Figure 14:**
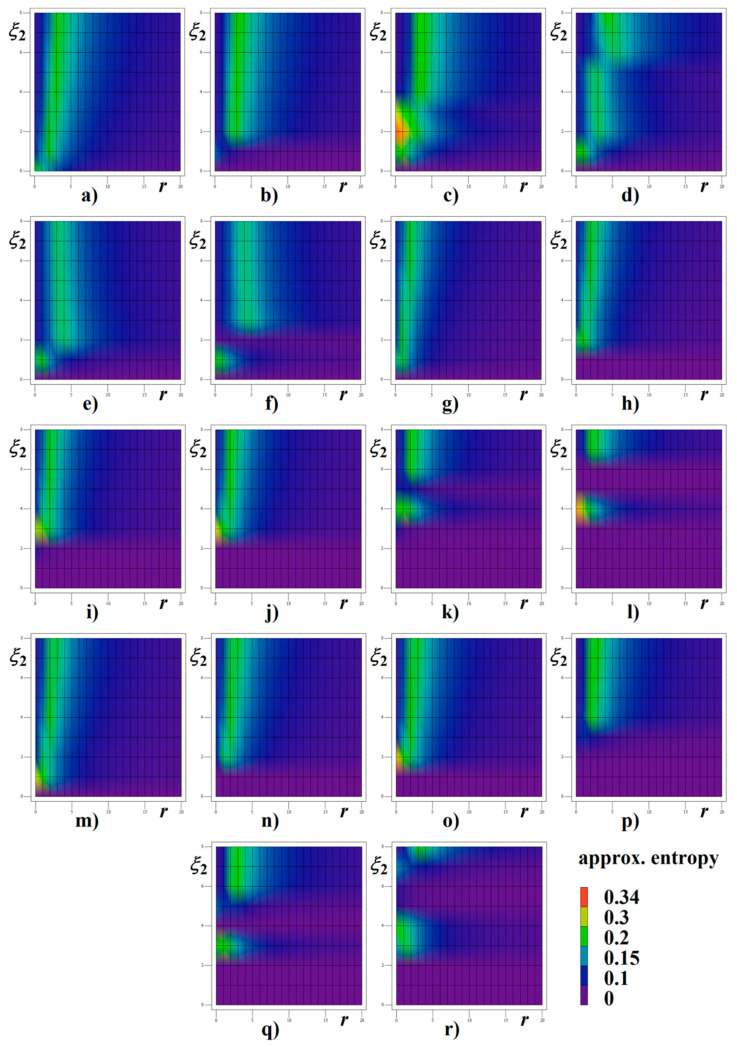
Approximate entropy calculated for memory with two CPEs of equivalent orders *α* = *β*, associated impedance norms *ξ*_1_, *ξ*_2_ and threshold *r*: (**a**) *α* = *β* = 8/9, *ξ*_1_ = 1, (**b**) *α* = *β* = 8/9, *ξ*_1_ = 2, (**c**) *α* = *β* = 8/9, *ξ*_1_ = 3, (**d**) *α* = *β* = 8/9, *ξ*_1_ = 4, (**e**) *α* = *β* = 8/9, *ξ*_1_ = 5, (**f**) *α* = *β* = 8/9, *ξ*_1_ = 6, (**g**) *α* = *β* = 4/5, *ξ*_1_ = 1, (**h**) *α* = *β* = 4/5, *ξ*_1_ = 2, (**i**) *α* = *β* = 4/5, *ξ*_1_ = 3, (**j**) *α* = *β* = 4/5, *ξ*_1_ = 4, (**k**) *α* = *β* = 4/5, *ξ*_1_ = 5, (**l**) *α* = *β* = 4/5, *ξ*_1_ = 6, (**m**) *α* = *β* = 7/9, *ξ*_1_ = 1, (**n**) *α* = *β* = 7/9, *ξ*_1_ = 2, (**o**) *α* = *β* = 7/9, *ξ*_1_ = 3, (**p**) *α* = *β* = 7/9, *ξ*_1_ = 4, (**q**) *α* = *β* = 7/9, *ξ*_1_ = 5, (**r**) *α* = *β* = 7/9, *ξ*_1_ = 6.

Both Figure 13 and Figure 14 demonstrate numerical investigation of systems (12) and (13) with respect to the entropic properties of the generated signals. The threshold *r* is the main parameter of the numerical algorithm, which measures and quantify similarity patterns in the data sequence of the increasing length (up to the self-comparison)—see tutorial paper [47] for a better understanding. In this picture, the rainbow color scale for ApEn quantity is utilized, see legend. Data for time integration of real circuit has been obtained from interval starting with 100 and ending with 200 ms. This data sequence clearly represents steady state of circuit with two CPEs.

**Figure 15:**
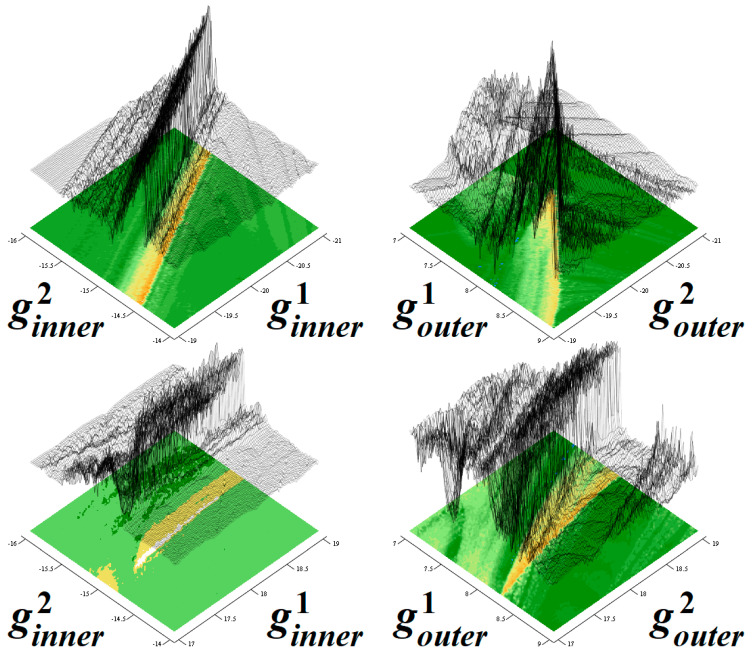
Topographically scaled surface-contour plot of LLE as a function of slopes of both PWL functions; high resolution is achieved by using the uniform parameter step 0.01.

Figure 15 provides graph of LLE as a function of slopes of both PWL functions. The minimum value of LLE is −0.09 and the maximum value is 0.153. The colored scale is as following: dark blue areas represent fixed point solution, green stands for limit cycle solutions, yellow and white denotes weak (LLE lower than 0.1) and strong (LLE greater than 0.1) chaos behavior. Since three-segment odd-symmetrical PWL functions∈ are considered for memory, this plot represents four-dimensional hypercube with edges *g*^1^_inner_∈(−21, −19), *g*^1^_outer_∈(7, 9), *g*^2^_inner_∈(−16, −14), *g*^2^_outer_∈(17, 19) and resolution 201 × 201 × 201 × 201 points. For this calculation, the fourth-order Runge-Kutta method in Matlab. Final time for integration was set to 1000 with transient behavior omitted.

**Figure 16:**
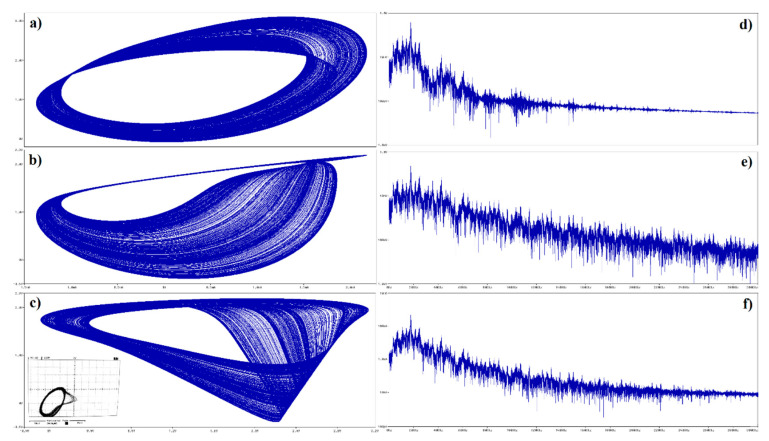
Orcad Pspice circuit simulation of IO memory in robust chaotic regime; plane projection: (**a**) *v_C_*_1_ vs. *i_L_*, (**b**) *v_C_*_2_ vs. *i_L_*, and (**c**) *v_C_*_2_ vs. *v_C_*_1_ together with the experiment; frequency spectrum of generated signal: (**d**) *v_C_*_1_, (**e**) *v_C_*_2_, and (**f**) *i_L_*.

**Figure 17:**
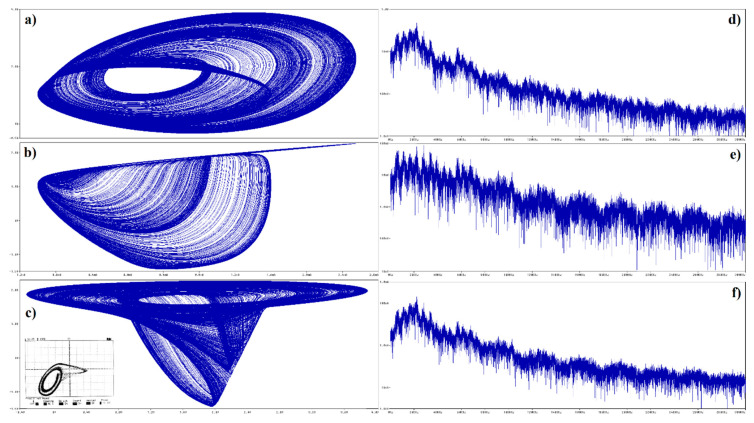
Orcad Pspice circuit simulation of FO memory of total mathematical order 2.7 working in robust chaotic regime; plane projection: (**a**) *v_C_*_1_ vs. *i_L_*, (**b**) *v_C_*_2_ vs. *i_L_*, and (**c**) *v_C_*_2_ vs. *v_C_*_1_ along with oscilloscope screenshot; frequency spectrum of generated signal: (**d**) *v_C_*_1_, (**e**) *v_C_*_2_, and (**f**) *i_L_*.

**Figure 18:**
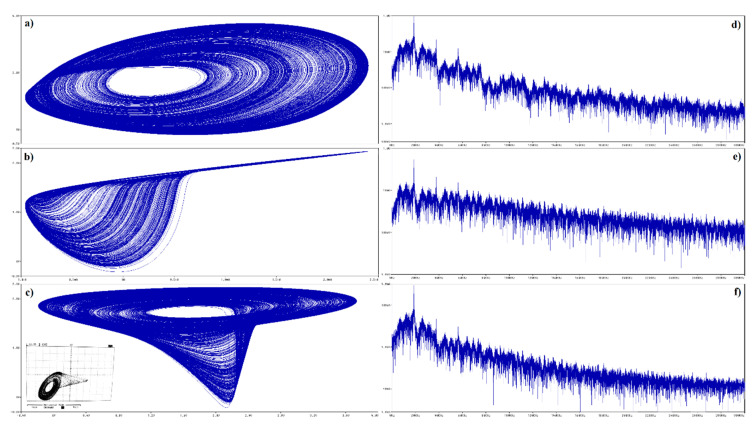
Orcad Pspice circuit simulation of FO memory of total mathematical order 2.4 working in robust chaotic regime; plane projection: (**a**) *v_C_*_1_ vs. *i_L_*, (**b**) *v_C_*_2_ vs. *i_L_*, and (**c**) *v_C_*_2_ vs. *v_C_*_1_ with experimental verification; frequency spectrum of generated signal: (**d**) *v_C_*_1_, (**e**) *v_C_*_2_, and (**f**) *i_L_*.

**Figure 19:**
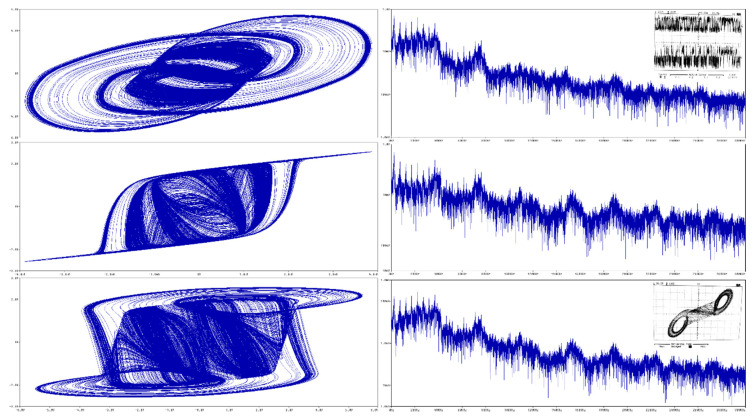
Orcad Pspice circuit simulation of FO memory of total mathematical order 2.4 working in the operation of a double-spiral generation; plane projection: (**a**) *v_C_*_1_ vs. *i_L_*, (**b**) *v_C_*_2_ vs. *i_L_* and (**c**) *v_C_*_2_ vs. *v_C_*_1_; frequency spectrum of generated signal: (**d**) *v_C_*_1_, (**e**) *v_C_*_2_, and (**f**) *i_L_*.

For practical experiments, fundamental frequency and impedance norm was chosen to be 10^5^ and 10^4^, respectively. Thus, real-valued integer-order capacitors are *C*_1_ = 10 nF, *C*_2_ = 6 nF and inductor is *L* = 10 mH. Remaining circuit components of this IO memory are *R_n_*_1_ = 3 k∧, *R_n_*_2_ = 1 k∧, *R_n_*_3_ = 180 ∧ and *R_n_*_4_ = 1.5 k∧. Computer-aided analysis of this dynamical system in the time domain is given in Figure 16. Initial conditions can be imposed into circuit by using pseudo-component IC1; it serves for the definition of the node voltage at the start of the time domain simulation. The same circuitry undergoes Orcad Pspice based simulation for two equivalent CPE having orders *α* = *β* = 9/10, see Figure 17 for brief results. While the value of the inductor was kept default, components of first and second CPE were adjusted by impedance norms 2 and 13, respectively. Of course, continuation with experiments can result into a circuit total order that is decreased even further. Such an example is given in Figure 18 where two equivalent CPEs are considered; each with math order *α* = *β* = 4/5. In this case, impedance norms were chosen to be equal to 15 and 55. Additionally, the famous double-scroll strange attractor can be generated by FO active memory. Corresponding proof can be found inside Figure 19 where both simulation and laboratory measurement are demonstrated. Here, original CPEs described in Section 3 of this paper were affected by impedance norms 3 and 16. For the above circuit calculations, parameters adjusted within simulation profile were set to the final time 50 ms, whereas maximum time step was decreased to 100 ns to obtain smooth state trajectories. It is worth nothing that these options guarantee good resolution for FFT calculation. True laboratory experiments are provided via oscilloscope screenshots located at bottom left corners of Figure 16, Figure 17, Figure 18 and within the right column in Figure 19. In the latter case, generated chaotic waveforms in time domain are also included.

## 6. Discussion

From the perspective of the reader, the orientation of this manuscript is towards three problems. Firstly, it brings thorough investigation of research and review papers focused on applications of FO circuits in analog design engineering (more precisely speaking, in area of continuous-time signal processing and generation). Considerable attention is paid on the various implementations of CPE. This section can help curious reader to find specific topic for their own research, to develop new application with promising properties, or to fix engineering problem unsolvable with IO circuits.

A wide spectrum of potential applications with wideband CPEs are proposed in Section 3 of this paper. These circuit elements can be used in the frequency filters, tunable harmonic oscillators and modeling of the complex dynamical systems derived by direct observations of nature phenomena. Series, parallel or a combination of series-parallel interconnection of resistors and capacitors are considered to reach values sufficiently close to those provided in the tables in Section 3 of this paper. These values can be transformed into locations of zeroes and poles (in the complex plane) of voltage and/or current transfer function for different kind of circuit realization of CPE. Knowledge of the mentioned positions itself can lead to cascade connection of bilinear filters, while coupling of zeroes and poles pairs result into cascade of biquadratic filtering two-ports. A systematic approach of how to use generalized band-pass and band-reject filtering section for CPE approximation is described in paper [48].

Each CPE provided in Section 3 undergoes tolerance analysis in Orcad Pspice, namely 1000 runs of Monte-Carlo (normal distribution for values of resistors and capacitors was applied) combined with standard AC sweep. As expected, passive components dedicated for the CPE design need to be very accurate. Larger fabrication tolerances such as 0.5%, 1%, or higher, are out of question for this purpose because it causes too large phase errors. Phase frequency response starts to be significantly rippled, nearby peaks and valleys of a phase pantile can sum-up leading to the maximal phase deviation significantly raised. Unfortunately, a higher phase difference between theoretical and obtained value occurs not locally, but globally, i.e., over an entire approximated frequency range. Practical experience resulting from survey of existing application-oriented research papers suggests that maximal phase error greater than 3° renders constructed CPE unserviceable. Moreover, individual orders become undistinguishable. Of course, usability of designed CPE always depends on concrete application. Even 1.5° maximal phase error can be too large for high-performance demanding applications.

## 7. Conclusions

This paper brings a rich gallery of high-precision CPEs dedicated for wideband signal processing. Readers can pick and use proposed CPEs directly, without the need of additional calculations. Individual designed CPEs have reasonable values of circuit components that can be found commonly in stocks of markets. Individual outputs resulting from this paper attract a wide spectrum of enthusiasts, electronic engineers, and design specialist to construct linear and nonlinear systems described by FO dynamics. Moreover, using designed CPEs, existing structures of FO filters, harmonic oscillators and arbitrary waveform generators (especially tunable in wide range), phase correctors, PID controllers, regulators, models of dynamical systems, etc. can be simulated again, and associated results can be polished.

From a nonlinear dynamics point of view, this paper demonstrates that FO analog memory can be chaotic, even if real CPEs are included both into mathematical model and real fabricated circuit. This is a new and so far unpublished reality, proved by means of numerical calculations, computer-aided analysis of memory circuit, as well as experimental outputs.

## Figures and Tables

**Table 1:** Approximation of CPE with order α = 1/10; RC series-parallel topology of fractal capacitor.

Rp/Cp	R_1_/C_1_	R_2_/C_2_	R_3_/C_3_	R_4_/C_4_	R_5_/C_5_	R_6_/C_6_	R_7_/C_7_
**596 Ω** 2.2 kΩ || 820 Ω	**2250 Ω** 2.2 kΩ ∠ 47 Ω	**1.8 kΩ** 1.8 kΩ	**1.4 kΩ** 1.2 kΩ ∠ 220 Ω	**1.1 kΩ** 1 kΩ ∠ 100 Ω	**884 Ω** 820 Ω ∠ 68 Ω	**699 Ω** 680 Ω ∠ 18 Ω	**553 Ω** 330 Ω ∠ 220 Ω
**20 pF** 10 pF || 10 pF	**44 μF** 22 μF || 22 μF	**5.4 μF** 3.3 nF || 2.2 nF	**653 nF** 470 nF || 180 nF	**79 nF** 47 nF || 33 nF	**9.6 nF** 8.2 nF || 1.5 nF	**1.2 nF** 1.2 nF	**142 pF** 120 pF || 22 pF

Bold represents desired value that can be reached by several ways.

**Table 2:** CPE with math order α = 1/10; fully passive RC parallel-series topology of fractal capacitor.

Rs/Cs	R_1_/C_1_	R_2_/C_2_	R_3_/C_3_	R_4_/C_4_	R_5_/C_5_	R_6_/C_6_	R_7_/C_7_
**93 kΩ** 82 kΩ ∠ 10 kΩ	**100 kΩ** 100 kΩ	**79 kΩ** 68 kΩ ∠ 10 kΩ	**63 kΩ** 47 kΩ ∠ 15 kΩ	**50 Ω** 100 kΩ || 100 kΩ	**39 kΩ** 39 kΩ	**31 kΩ** 27 kΩ ∠ 3.9 kΩ	**24.5 kΩ** 15 kΩ ∠ 10 kΩ
**7.24 μF** 3.9 μF || 3.3 μF	**1 μF** 1 μF	**121 nF** 120 nF || 1 nF	**14.7 nF** 12 nF || 2.7 nF	**1.8 nF** 1.8 nF	**217 pF** 15 nF ∠ 220 pF	**26 pF** 22 pF || 3.9 pF	**3.4 pF** 2.2 pF || 1.2 pF

**Table 3:** Approximation of CPE with order α = 1/9; RC series-parallel topology of fractal capacitor.

Rp/Cp	R_1_/C_1_	R_2_/C_2_	R_3_/C_3_	R_4_/C_4_	R_5_/C_5_	R_6_/C_6_	R_7_/C_7_
**6.3 kΩ** 4.7 kΩ ∠ 1.5 kΩ	**21.3 kΩ** 18 kΩ ∠ 3.3 kΩ	**16.4 kΩ** 15 kΩ ∠ 1.5 kΩ	**12.6 kΩ** 12 kΩ ∠ 560 Ω	**9.7 kΩ** 8.2 kΩ ∠ 1.5 Ω	**7.5 kΩ** 15 kΩ || 15 kΩ	**5.8 kΩ** 5.6 kΩ ∠ 220 Ω	**4.5 kΩ** 3.9 kΩ ∠ 560 Ω
**2.5 pF** 1 pF || 1.5 pF	**4.7 μF** 4.7 μF	**586 nF** 560 nF || 27 nF	**73 nF** 68 nF || 4.7 nF	**9.1 nF** 8.2 nF || 1 nF	**1.1 nF** 1 nF || 100 pF	**141 pF** 120 pF || 22 pF	**17.6 pF** 15 pF || 2.7 pF

**Table 4:** CPE with math order α = 1/9; fully passive RC parallel-series topology of fractal capacitor.

Rs/Cs	R_1_/C_1_	R_2_/C_2_	R_3_/C_3_	R_4_/C_4_	R_5_/C_5_	R_6_/C_6_	R_7_/C_7_
**70.5 kΩ** 68 kΩ ∠ 2.2 kΩ	**100 kΩ** 100 kΩ	**77 kΩ** 39 kΩ ∠ 39 kΩ	**59.4 kΩ** 56 kΩ ∠ 3.3 kΩ	**45.8 kΩ** 39 kΩ ∠ 6.8 kΩ	**35.3 kΩ** 33 kΩ ∠ 2.2 kΩ	**27.2 kΩ** 27 kΩ ∠ 220 Ω	**21 kΩ** 15 kΩ ∠ 5.6 kΩ
**7 μF** 6.8 μF || 220 nF	**1 μF** 1 μF	**124.5 nF** 120 nF || 4.7 nF	**15.5 nF** 10 nF || 5.6 nF	**1.9 nF** 1.8 nF || 100 pF	**241 pF** 220 pF || 22 pF	**30 pF** 15 pF || 15 pF	**3.9 pF** 3.9 pF

**Table 5:** Approximation of CPE with order α = 1/5; RC series-parallel topology of fractal capacitor.

Rp/Cp	R_1_/C_1_	R_2_/C_2_	R_3_/C_3_	R_4_/C_4_	R_5_/C_5_	R_6_/C_6_	R_7_/C_7_
**8.2 kΩ** 8.2 kΩ	**12.7 kΩ** 10 kΩ ∠ 2.7 kΩ	**8.6 kΩ** 8.6 kΩ	**5.4 kΩ** 3.9 kΩ ∠ 1.5 kΩ	**3364 Ω** 3.3 kΩ ∠ 68 Ω	**2.1 kΩ** 47 kΩ || 2.2 kΩ	**1320 Ω** 1.2 kΩ ∠ 120 Ω	**825 Ω** 820 Ω ∠ 4.7 Ω
**17.2 pF** 15 pF || 2.2 pF	**7.3 μF** 27 μF ∠ 10 μF	**1.1 μF** 1 μF || 100 nF	**171 nF** 150 nF || 22 nF	**26 nF** 22 nF || 3.9 nF	**4 nF** 3.9 nF || 100 pF	**619 pF** 6.8 nF ∠ 680 pF	**95 pF** 82 pF || 12 pF

**Table 6:** CPE with math order α = 1/5; fully passive RC parallel-series topology of fractal capacitor.

Rs/Cs	R_1_/C_1_	R_2_/C_2_	R_3_/C_3_	R_4_/C_4_	R_5_/C_5_	R_6_/C_6_	R_7_/C_7_
**10 kΩ** 10 kΩ	**100 kΩ** 100 kΩ	**62.6 kΩ** 270 kΩ || 82 kΩ	**39 kΩ** 39 kΩ	**24.5 kΩ** 15 kΩ ∠ 10 kΩ	**15.3 kΩ** 15 kΩ ∠ 270 Ω	**9.6 kΩ** 8.6 kΩ ∠ 1 kΩ	**6 kΩ** 12 kΩ || 12 kΩ
**5.5 μF** 4.7 μF || 820 nF	**1 μF** 1 μF	**153 nF** 150 nF || 3.3 nF	**23.5 nF** 22 nF || 1.5 nF	**3.6 nF** 1.8 nF || 1.8 nF	**554 pF** 470 pF || 82 pF	**85 pF** 82 pF || 3.3 pF	**15 pF** 15 pF

**Table 7:** Approximation of CPE with order α = 2/9; RC series-parallel topology of fractal capacitor.

Rp/Cp	R_1_/C_1_	R_2_/C_2_	R_3_/C_3_	R_4_/C_4_	R_5_/C_5_	R_6_/C_6_	R_7_/C_7_
**8.4 kΩ** 8.2 kΩ ∠ 220 Ω	**12.2 kΩ** 12 kΩ ∠ 220 Ω	**7.7 kΩ** 120 kΩ || 8.2 kΩ	**4670 Ω** 27 kΩ || 5.6 kΩ	**2.7 kΩ** 2.7 kΩ	**1.7 kΩ** 1.5 kΩ ∠ 220 Ω	**1 kΩ** 1 kΩ	**626 Ω** 8.2 kΩ || 680 Ω
**43 pF** 39 pF || 3.9 pF	**7.8 μF** 6.8 μF || 1 μF	**1.35 μF** 1.2 μF || 150 nF	**233 nF** 220 nF || 12 nF	**40 nF** 82 nF ∠ 82 nF	**6.8 nF** 6.8 nF	**1.2 nF** 1.2 nF	**206 pF** 180 pF || 27 pF

**Table 8:** CPE with math order α = 2/9; fully passive RC parallel-series topology of fractal capacitor.

Rs/Cs	R_1_/C_1_	R_2_/C_2_	R_3_/C_3_	R_4_/C_4_	R_5_/C_5_	R_6_/C_6_	R_7_/C_7_
**7.5 kΩ** 15 kΩ || 15 kΩ	**100 kΩ** 100 kΩ	**60.5 kΩ** 39 kΩ ∠ 22 kΩ	**36.6 kΩ** 33 kΩ ∠ 3.3 kΩ	**22 kΩ** 22 kΩ	**13.4 kΩ** 12 kΩ ∠ 150 Ω	**8.2 kΩ** 8.2 kΩ	**5.2 kΩ** 82 kΩ || 5.6 kΩ
**4.8 μF** 4.7 μF || 100 nF	**1 μF** 1 μF	**172 nF** 150 nF || 22 nF	**29.7 nF** 27 nF || 2.7 nF	**5.1 nF** 4.7 nF || 390 pF	**884 pF** 820 pF || 68 pF	**152 pF** 150 pF || 2.2 pF	**27 pF** 27 pF

**Table 9:** Approximation of CPE with order α = 1/4; RC series-parallel topology of fractal capacitor.

Rp/Cp	R_1_/C_1_	R_2_/C_2_	R_3_/C_3_	R_4_/C_4_	R_5_/C_5_	R_6_/C_6_	R_7_/C_7_
**8.5 kΩ** 4.7 kΩ ∠ 3.9 kΩ	**11.5 kΩ** 10 kΩ ∠ 1.5 kΩ	**6.6 kΩ** 3.3 kΩ ∠ 3.3 kΩ	**3.8 kΩ** 2.2 kΩ ∠ 1.5 kΩ	**2.2 kΩ** 2.2 kΩ	**1256 Ω** 1.2 kΩ ∠ 56 Ω	**722 Ω** 680 Ω ∠ 47 Ω	**415 Ω** 390 Ω ∠ 27 Ω
**95 pF** 82 pF || 12 pF	**8.2 μF** 8.2 μF	**1.65 μF** 1.5 μF || 150 nF	**313 nF** 270 nF || 47 nF	**59.4 nF** 56 nF || 3.3 nF	**11.3 nF** 10 nF || 1.2 nF	**2.1 nF** 10 nF ∠ 2.7 nF	**406 pF** 390 pF || 15 pF

**Table 10:** CPE with math order α = 1/4; fully passive RC parallel-series topology of fractal capacitor.

Rs/Cs	R_1_/C_1_	R_2_/C_2_	R_3_/C_3_	R_4_/C_4_	R_5_/C_5_	R_6_/C_6_	R_7_/C_7_
**4870 Ω** 4.7 kΩ ∠ 180 Ω	**100 kΩ** 100 kΩ	**57.5 kΩ** 56 kΩ ∠ 1.5 kΩ	**33 kΩ** 33 kΩ	**19 kΩ** 18 kΩ ∠ 1 kΩ	**11 kΩ** 10 kΩ ∠ 1 kΩ	**6270 Ω** 5.6 kΩ ∠ 680 Ω	**3.9 kΩ** 3.9 kΩ
**4.3 μF** 3.3 μF || 1 μF	**1 μF** 1 μF	**190 nF** 180 nF || 10 nF	**36 nF** 33 nF || 2.7 nF	**6.8 nF** 6.8 nF	**1.3 nF** 1.2 nF || 100 pF	**246 pF** 220 pF || 27 pF	**47 pF** 47 pF

**Table 11:** Approximation of CPE with order α = 3/10; RC series-parallel topology of fractal capacitor.

Rp/Cp	R_1_/C_1_	R_2_/C_2_	R_3_/C_3_	R_4_/C_4_	R_5_/C_5_	R_6_/C_6_	R_7_/C_7_
**9 kΩ** 18 kΩ || 18 kΩ	**8.6 kΩ** 8.2 kΩ ∠ 390 Ω	**4.9 kΩ** 3.9 kΩ ∠ 1 kΩ	**2.5 kΩ** 1.5 kΩ ∠ 1 kΩ	**1.3 kΩ** 1.2 kΩ ∠ 100 Ω	**665 Ω** 27 kΩ || 680 Ω	**342 Ω** 330 Ω ∠ 12 Ω	**180 Ω** 180 Ω
**258 pF** 220 pF || 39 pF	**10.5 μF** 10 μF || 470 nF	**2.2 μF** 2.2 μF	**474 nF** 470 nF || 3.9 nF	**100 nF** 100 nF	**21.3 nF** 18 nF || 3.3 nF	**4.5 nF** 3.3 nF || 1.2 nF	**1 nF** 1 nF

**Table 12:** CPE with math order α = 3/10; fully passive RC parallel-series topology of fractal capacitor.

Rs/Cs	R_1_/C_1_	R_2_/C_2_	R_3_/C_3_	R_4_/C_4_	R_5_/C_5_	R_6_/C_6_	R_7_/C_7_
**2 kΩ** 1 kΩ ∠ 1 kΩ	**100 kΩ** 100 kΩ	**51.4 kΩ** 220 kΩ || 68 kΩ	**26.5 kΩ** 82 kΩ || 39 kΩ	**13.6 kΩ** 12 kΩ ∠ 1.5 kΩ	**7 kΩ** 6.8 kΩ ∠ 220 Ω	**3.6 kΩ** 1.8 kΩ ∠ 1.8 kΩ	**2 kΩ** 1 kΩ ∠ 1 kΩ
**3.7 μF** 3.3 μF || 390 nF	**1 μF** 1 μF	**212 nF** 180 nF || 33 nF	**45 nF** 33 nF || 12 nF	**9.5 nF** 8.2 nF || 1.2 nF	**2 nF** 1 nF || 1 nF	**429 pF** 390 pF || 39 pF	**91 pF** 82 pF || 10 pF

**Table 13:** Approximation of CPE with order α = 1/3; RC series-parallel topology of fractal capacitor.

Rp/Cp	R_1_/C_1_	R_2_/C_2_	R_3_/C_3_	R_4_/C_4_	R_5_/C_5_	R_6_/C_6_	R_7_/C_7_
**9.2 kΩ** 8.2 kΩ ∠ 1 kΩ	**7.9 kΩ** 220 kΩ || 8.2 kΩ	**4 kΩ** 3.9 kΩ ∠ 100 Ω	**1920 Ω** 1.8 kΩ ∠ 120 Ω	**920 Ω** 820 Ω ∠ 100 Ω	**439 Ω** 390 Ω ∠ 47 Ω	**210 Ω** 4.7 kΩ || 220 Ω	**100 Ω** 100 Ω
**497 pF** 470 pF || 27 pF	**12 μF** 12 μF	**2.7 μF** 2.7 μF	**618 nF** 6.8 μF ∠ 680 nF	**141 nF** 120 nF || 22 nF	**32.2 nF** 180 nF ∠ 39 nF	**7.4 nF** 15 nF ∠ 15 nF	**1.8 nF** 1.8 nF

**Table 14:** CPE with math order α = 1/3; fully passive RC parallel-series topology of fractal capacitor.

Rs/Cs	R_1_/C_1_	R_2_/C_2_	R_3_/C_3_	R_4_/C_4_	R_5_/C_5_	R_6_/C_6_	R_7_/C_7_
**1100 Ω** 1 kΩ ∠ 100 Ω	**100 kΩ** 100 kΩ	**47.8 kΩ** 47 kΩ ∠ 820 Ω	**22.8 kΩ** 22 kΩ ∠ 820 Ω	**11 kΩ** 10 kΩ ∠ 1 kΩ	**5.2 kΩ** 4.7 kΩ ∠ 470 Ω	**2.5 kΩ** 1.5 kΩ ∠ 1 kΩ	**1.2 kΩ** 1.2 kΩ
**3.38 μF** 3.3 μF || 82 nF	**1 μF** 1 μF	**228 nF** 220 nF || 8.2 nF	**52 nF** 47 nF || 4.7 nF	**12 nF** 12 nF	**2.7 nF** 2.7 nF	**620 pF** 6.8 nF ∠ 80 pF	**155 pF** 100 pF || 56 pF

**Table 15:** Approximation of CPE with order α = 2/5; RC series-parallel topology of fractal capacitor.

Rp/Cp	R_1_/C_1_	R_2_/C_2_	R_3_/C_3_	R_4_/C_4_	R_5_/C_5_	R_6_/C_6_	R_7_/C_7_
**9430 Ω** 8.2 kΩ ∠ 1.2 kΩ	**6.8 kΩ** 6.8 kΩ	**3030 Ω** 2.7 kΩ ∠ 330 Ω	**1.3 kΩ** 1.2 kΩ ∠ 100 Ω	**555 Ω** 470 Ω ∠ 82 Ω	**238 Ω** 220 Ω ∠ 18 Ω	**102 Ω** 100 Ω ∠ 2.2 Ω	**47 Ω** 47 Ω
**2.7 nF** 2.7 nF	**13 μF** 12 μF || 1 μF	**4 μF** 3.9 μF || 100 nF	**1.1 μF** 1 μF || 100 nF	**311 nF** 5.6 μF ∠ 330 nF	**87.3 nF** 680 nF ∠ 100 nF	**24.5 nF** 270 nF ∠ 27 nF	**6.8 nF** 6.8 nF

**Table 16:** CPE with math order α = 2/5; fully passive RC parallel-series topology of fractal capacitor.

Rs/Cs	R_1_/C_1_	R_2_/C_2_	R_3_/C_3_	R_4_/C_4_	R_5_/C_5_	R_6_/C_6_	R_7_/C_7_
**470 Ω** 470 Ω	**100 kΩ** 100 kΩ	**42.8 kΩ** 470 kΩ || 47 kΩ	**18.3 kΩ** 15 kΩ ∠ 3.3 kΩ	**7850 Ω** 180 kΩ || 8.2 kΩ	**3360 Ω** 3.3 kΩ ∠ 56 Ω	**1420 Ω** 1.2 kΩ ∠ 220 Ω	**617 Ω** 6.8 kΩ || 680 Ω
**1.89 μF** 1.5 μF || 390 nF	**665 nF** 560 nF || 10 nF	**196 nF** 180 nF || 15 nF	**55 nF** 39 nF || 15 nF	**15.4 nF** 15 nF || 390 pF	**4.34 nF** 3.3 nF || 1 nF	**1.2 nF** 1.2 nF	**392 pF** 390 pF

**Table 17:** Approximation of CPE with order α = 4/9; RC series-parallel topology of fractal capacitor.

Rp/Cp	R_1_/C_1_	R_2_/C_2_	R_3_/C_3_	R_4_/C_4_	R_5_/C_5_	R_6_/C_6_	R_7_/C_7_
**9120 Ω** 39 kΩ || 12 kΩ	**5440 Ω** 180 kΩ || 5.6 kΩ	**2030 Ω** 1.8 kΩ ∠ 220 Ω	**759 Ω** 680 Ω ∠ 82 Ω	**284 Ω** 270 Ω ∠ 15 Ω	**106 Ω** 68 Ω ∠ 39 Ω	**40 Ω** 82 Ω || 82 Ω	**15 Ω** 15 Ω
**3.8 nF** 2.7 nF || 1 nF	**13.9 μF** 10 μF || 3.9 μF	**4.3 μF** 3.3 μF || 1 μF	**1.26 μF** 1 μF || 270 nF	**366 nF** 330 nF || 39 nF	**107 nF** 100 nF || 6.8 nF	**31.3 nF** 22 nF || 10 nF	**9.5 nF** 8.2 nF || 1.2 nF

**Table 18:** CPE with math order α = 4/9; fully passive RC parallel-series topology of fractal capacitor.

Rs/Cs	R_1_/C_1_	R_2_/C_2_	R_3_/C_3_	R_4_/C_4_	R_5_/C_5_	R_6_/C_6_	R_7_/C_7_
**130 Ω** 120 Ω ∠ 10 Ω	**72 kΩ** 68 kΩ ∠ 3.9 kΩ	**30 kΩ** 15 kΩ ∠ 15 kΩ	**11 kΩ** 10 kΩ ∠ 1 kΩ	**4170 Ω** 39 kΩ || 4.7 kΩ	**1560 Ω** 1.5 kΩ ∠ 56 Ω	**582 Ω** 560 Ω ∠ 22 Ω	**220 Ω** 220 Ω
**2.4 μF** 1.2 μF || 1.2 μF	**1 μF** 1 μF	**292 nF** 270 nF || 22 nF	**85.3 nF** 82 nF || 3.3 nF	**24.9 nF** 15 nF || 10 nF	**7.3 nF** 68 nF ∠ 8.2 nF	**2.1 nF** 47 nF ∠ 2.2 nF	**680 pF** 680 pF

**Table 19:** Approximation of CPE with order α = 1/2; RC series-parallel topology of fractal capacitor.

Rp/Cp	R_1_/C_1_	R_2_/C_2_	R_3_/C_3_	R_4_/C_4_	R_5_/C_5_	R_6_/C_6_	R_7_/C_7_
**9490 Ω** 180 kΩ ∠ 10 kΩ	**4680 Ω** 27 kΩ || 5.6 kΩ	**1550 Ω** 1.5 kΩ ∠ 47 Ω	**510 Ω** 470 Ω ∠ 39 Ω	**169 Ω** 100 Ω ∠ 68 Ω	**56 Ω** 56 Ω	**18 Ω** 18 Ω	**6.4 Ω** 4.7 Ω ∠ 1.8 Ω
**11 nF** 10 nF || 1 nF	**16 μF** 15 μF || 1 μF	**5.65 μF** 4.7 μF || 1 μF	**1.87 μF** 1.5 μF || 390 nF	**616 nF** 560 nF || 56 nF	**204 nF** 150 nF || 56 nF	**67.2 nF** 390 nF ∠ 82 nF	**21.4 nF** 18 nF || 3.3 nF

**Table 20:** CPE with math order α = 1/2; fully passive RC parallel-series topology of fractal capacitor.

Rs/Cs	R_1_/C_1_	R_2_/C_2_	R_3_/C_3_	R_4_/C_4_	R_5_/C_5_	R_6_/C_6_	R_7_/C_7_
**51.2 Ω** 680 Ω || 56 Ω	**75 kΩ** 150 kΩ || 150 kΩ	**26.4 kΩ** 18 kΩ ∠ 8.2 kΩ	**8730 Ω** 68 kΩ || 10 kΩ	**2880 Ω** 2.7 kΩ ∠ 180 Ω	**952 Ω** 680 Ω ∠ 270 Ω	**314.5 Ω** 6.8 kΩ || 330 Ω	**104 Ω** 100 Ω ∠ 3.9 Ω
**2 μF** 1 μF || 1 μF	**980 nF** 820 nF || 150 nF	**330 nF** 330 nF	**109nF** 100 nF || 10 nF	**36 nF** 22 nF || 15 nF	**12 nF** 12 nF	**3.9 nF** 3.9 nF	**1.39 nF** 1 nF || 390 pF

**Table 21:** Approximation of CPE with order α = 5/9; RC series-parallel topology of fractal capacitor.

Rp/Cp	R_1_/C_1_	R_2_/C_2_	R_3_/C_3_	R_4_/C_4_	R_5_/C_5_	R_6_/C_6_	R_7_/C_7_
**10 kΩ** 10 kΩ	**4110 Ω** 3.9 kΩ ∠ 220 Ω	**1.2 kΩ** 1.2 kΩ	**350 Ω** 330 Ω ∠ 22 Ω	**102 Ω** 100 Ω ∠ 2.2 Ω	**30 Ω** 15 Ω ∠ 15 Ω	**9 Ω** 18 Ω || 18 Ω	**2.7 Ω** 2.7 Ω
**31.55 nF** 22 nF || 10 nF	**18 μF** 18 μF	**7.3 μF** 4.7 μF || 2.7 μF	**2.7 μF** 2.7 μF	**1 μF** 1 μF	**379 nF** 330 nF || 47 nF	**144 nF** 120 nF || 22 nF	**56 nF** 56 nF

**Table 22:** CPE with math order α = 5/9; fully passive RC parallel-series topology of fractal capacitor.

Rs/Cs	R_1_/C_1_	R_2_/C_2_	R_3_/C_3_	R_4_/C_4_	R_5_/C_5_	R_6_/C_6_	R_7_/C_7_
**20.5 Ω** 10 Ω ∠ 10 Ω	**75 kΩ** 150 kΩ || 150 kΩ	**23.4 kΩ** 22 kΩ ∠ 1.5 kΩ	**6820 Ω** 6.8 kΩ	**2 kΩ** 1 kΩ ∠ 1 kΩ	**582 Ω** 560 Ω ∠ 22 Ω	**170 Ω** 100 Ω ∠ 68 Ω	**50 Ω** 100 Ω || 100 Ω
**1.7 μF** 1 μF || 680 nF	**1 μF** 1 μF	**374 nF** 1.8 μF ∠ 470 nF	**140nF** 100 nF || 39 nF	**52 nF** 33 nF || 18 nF	**19.5 nF** 18 nF || 1.5 nF	**7.3 nF** 6.8 nF || 470 pF	**2.9 nF** 2.2 nF || 680 pF

**Table 23:** Approximation of CPE with order α = 3/5; RC series-parallel topology of fractal capacitor.

Rp/Cp	R_1_/C_1_	R_2_/C_2_	R_3_/C_3_	R_4_/C_4_	R_5_/C_5_	R_6_/C_6_	R_7_/C_7_
**8790 Ω** 68 kΩ || 10 kΩ	**3160 Ω** 68 kΩ || 3.3 kΩ	**837 Ω** 820 Ω ∠ 18 Ω	**221.5 Ω** 220 Ω ∠ 1.5 Ω	**58.6 Ω** 56 kΩ ∠ 2.7 kΩ	**15.5 Ω** 15 Ω	**4.1 Ω** 8.2 Ω || 8.2 Ω	**1.2 Ω** 1.2 Ω
**65.3 nF** 47 nF || 18 nF	**18 μFμ** 18 μF	**7.8 μF** 5.6 nF || 2.2 nF	**3.2 μF** 2.7 μF || 470 nF	**1.3 μF** 1 μF || 100 nF	**548 nF** 330 nF || 220 nF	**226 nF** 220 nF || 5.6 nF	**93 nF** 82 nF || 10 nF

**Table 24:** CPE with math order α = 3/5; fully passive RC parallel-series topology of fractal capacitor.

Rs/Cs	R_1_/C_1_	R_2_/C_2_	R_3_/C_3_	R_4_/C_4_	R_5_/C_5_	R_6_/C_6_	R_7_/C_7_
**7.4 Ω** 5.6 Ω ∠ 1.8 Ω	**53 kΩ** 39 kΩ ∠ 15 kΩ	**15.9 kΩ** 12 kΩ ∠ 3.9 kΩ	**4.2 kΩ** 3.9 kΩ || 330 Ω	**1.1 kΩ** 1 kΩ ∠ 100 Ω	**294 Ω** 220 Ω ∠ 82 Ω	**78 Ω** 68 Ω ∠ 10 Ω	**20.6 Ω** 15 Ω ∠ 5.6 Ω
**1.4 μF** 1 μF ∠ 390 nF	**1 μF** 1 μF	**412 nF** 390 nF || 22 nF	**170 nF** 100 nF || 68 nF	**70 nF** 47 nF || 22 nF	**28.9 nF** 27 nF || 1.8 nF	**11.9 nF** 10 nF || 1.8 nF	**5.2 nF** 3.9 nF || 1.2 nF

**Table 25:** Approximation of CPE with order α = 2/3; RC series-parallel topology of fractal capacitor.

Rp/Cp	R_1_/C_1_	R_2_/C_2_	R_3_/C_3_	R_4_/C_4_	R_5_/C_5_	R_6_/C_6_	R_7_/C_7_
**900 kΩ** 1.8 MΩ || 1.8 MΩ	**288 kΩ** 270 kΩ ∠ 18 kΩ	**70.2 kΩ** 68 kΩ ∠ 2.2 kΩ	**17.1 kΩ** 15 kΩ ∠ 2.2 Ω	**4150 Ω** 3.9 kΩ ∠ 220 Ω	**1010 Ω** 1 kΩ ∠ 10 Ω	**246 Ω** 220 Ω ∠ 27 Ω	**60 Ω** 120 Ω || 120 Ω
**2.9 nF** 2.7 nF || 220 pF	**190 nF** 180 nF || 10 nF	**103 nF** 82 nF || 22 nF	**50.6 nF** 33 nF || 18 nF	**25 nF** 15 nF || 10 nF	**12.3 nF** 10 nF || 2.2 nF	**6.1 nF** 12 nF ∠ 12 nF	**3.3 nF** 3.3 nF

**Table 26:** CPE with math order α = 2/3; fully passive RC parallel-series topology of fractal capacitor.

Rs/Cs	R_1_/C_1_	R_2_/C_2_	R_3_/C_3_	R_4_/C_4_	R_5_/C_5_	R_6_/C_6_	R_7_/C_7_
**40 Ω** 39 Ω ∠ 1 Ω	**560 kΩ** 560 kΩ	**146 kΩ** 100 kΩ ∠ 47 kΩ	**35.5 kΩ** 18 kΩ ∠ 18 kΩ	**8640 Ω** 6.8 kΩ ∠ 1.8 kΩ	**2.1 kΩ** 47 kΩ || 2.2 kΩ	**511 Ω** 390 Ω ∠ 120 Ω	**124 Ω** 120 Ω ∠ 3.9 Ω
**103 nF** 82 nF || 22 nF	**95 nF** 82 nF ∠ 12 nF	**49.3 nF** 47 nF || 2.2 nF	**24.3 nF** 22 nF || 2.2 nF	**12 nF** 12 nF	**5.9 nF** 5.6 nF || 330 pF	**2.9 nF** 2.7 nF || 220 pF	**1.6 nF** 1.5 nF || 100 pF

**Table 27:** Approximation of CPE with order α = 7/10; RC series-parallel topology of fractal capacitor.

Rp/Cp	R_1_/C_1_	R_2_/C_2_	R_3_/C_3_	R_4_/C_4_	R_5_/C_5_	R_6_/C_6_	R_7_/C_7_
**820 kΩ** 820 kΩ	**240 kΩ** 220 kΩ ∠ 22 kΩ	**54.6 kΩ** 33 kΩ ∠ 22 kΩ	**12.4 kΩ** 12 kΩ ∠ 390 Ω	**2.8 kΩ** 2.7 kΩ ∠ 100 Ω	**636 Ω** 10 kΩ || 680 Ω	**144 Ω** 120 Ω ∠ 22 Ω	**33 Ω** 33 Ω
**5.1 nF** 4.7 nF || 390 pF	**200 nF** 100 nF || 100 nF	**110 nF** 100 nF || 10 nF	**58.2 nF** 56 nF || 2.2 nF	**30.8 nF** 27 nF || 3.9 nF	**16.3 nF** 15 nF || 1.2 nF	**8.6 nF** 8.2 nF || 390 pF	**4.8 nF** 4.7 nF || 100 pF

**Table 28:** CPE with math order α = 7/10; fully passive RC parallel-series topology of fractal capacitor.

Rs/Cs	R_1_/C_1_	R_2_/C_2_	R_3_/C_3_	R_4_/C_4_	R_5_/C_5_	R_6_/C_6_	R_7_/C_7_
**20 Ω** 10 Ω ∠ 10 Ω	**500 kΩ** 1 MΩ || 1 MΩ	**113 kΩ** 100 kΩ ∠ 12 kΩ	**25.7 kΩ** 22 kΩ ∠ 3.9 kΩ	**5820 Ω** 5.6 kΩ ∠ 220 Ω	**1.3 kΩ** 1.2 kΩ ∠ 100 Ω	**300 Ω** 150 Ω ∠ 150 Ω	**68 Ω** 68 Ω
**90 nF** 180 nF ∠ 180 nF	**90 nF** 180 nF ∠ 180 nF	**52.9 nF** 1 μF ∠ 56 nF	**28 nF** 27 nF || 1 nF	**14.8 nF** 12 nF || 2.7 nF	**7.9 nF** 4.7 nF || 3.3 nF	**4.2 nF** 2.7 nF || 1.5 nF	**2.4 nF** 1.2 nF || 1.2 nF

**Table 29:** Approximation of CPE with order α = 3/4; RC series-parallel topology of fractal capacitor.

Rp/Cp	R_1_/C_1_	R_2_/C_2_	R_3_/C_3_	R_4_/C_4_	R_5_/C_5_	R_6_/C_6_	R_7_/C_7_
**872 kΩ** 6.8 MΩ || 1 MΩ	**223 kΩ** 220 kΩ ∠ 3.3 kΩ	**45.6 kΩ** 39 kΩ ∠ 6.8 kΩ	**9290 Ω** 8.2 kΩ ∠ 82 Ω	**1.9 kΩ** 1.8 kΩ ∠ 100 Ω	**386 Ω** 330 Ω ∠ 56 Ω	**79 Ω** 39 Ω ∠ 39 Ω	**18 Ω** 18 Ω
**13.3 nF** 10 nF || 3.3 nF	**210 nF** 4.7 μF ∠ 220 nF	**132 nF** 100 nF || 33 nF	**77.5 nF** 39 nF || 39 nF	**45.6 nF** 39 nF || 6.8 nF	**27 nF** 27 nF	**15.8 nF** 15 nF || 820 pF	**10 nF** 10 nF

**Table 30:** CPE with math order α = 3/4; fully passive RC parallel-series topology of fractal capacitor.

Rs/Cs	R_1_/C_1_	R_2_/C_2_	R_3_/C_3_	R_4_/C_4_	R_5_/C_5_	R_6_/C_6_	R_7_/C_7_
**9.2 Ω** 8.2 Ω ∠ 1 Ω	**470 kΩ** 470 kΩ	**102 kΩ** 100 kΩ ∠ 2.2 kΩ	**20.8 kΩ** 18 kΩ || 2.7 kΩ	**4240 Ω** 3.9 kΩ ∠ 330 Ω	**864 Ω** 820 Ω ∠ 47 Ω	**176 Ω** 150 Ω ∠ 27 Ω	**36 Ω** 18 Ω ∠ 18 Ω
**70 nF** 470 nF ∠ 82 nF	**90 nF** 180 nF ∠ 180 nF	**59 nF** 47 nF || 12 nF	**34.6 nF** 33 nF || 1.5 nF	**20.4 nF** 15 nF || 5.6 nF	**12 nF** 12 nF	**7.1 nF** 5.6 nF || 1.5 nF	**4.2 nF** 8.2 nF ∠ 8.2 nF

**Table 31:** Approximation of CPE with order α = 7/9; RC series-parallel topology of fractal capacitor.

Rp/Cp	R_1_/C_1_	R_2_/C_2_	R_3_/C_3_	R_4_/C_4_	R_5_/C_5_	R_6_/C_6_	R_7_/C_7_
**1.1 MΩ** 1 MΩ ∠ 100 kΩ	**240 kΩ** 120 kΩ ∠ 120 kΩ	**43 kΩ** 39 kΩ ∠ 3.9 kΩ	**7.7 kΩ** 6.8 kΩ ∠ 1 kΩ	**1374 Ω** 1.2 kΩ ∠ 180 Ω	**245 Ω** 180 Ω ∠ 68 Ω	**44 Ω** 22 Ω ∠ 22 Ω	**7.8 Ω** 6.8 Ω ∠ 1 Ω
**20 nF** 10 nF || 10 nF	**240 nF** 120 nF || 120 nF	**152 nF** 120 nF || 33 nF	**93 nF** 82 nF || 10 nF	**57 nF** 47 nF || 10 nF	**34.6 nF** 33 nF || 1.5 nF	**21.2 nF** 18 nF || 3.3 nF	**14 nF** 10 nF || 3.9 nF

**Table 32:** CPE with math order α = 7/9; fully passive RC parallel-series topology of fractal capacitor.

Rs/Cs	R_1_/C_1_	R_2_/C_2_	R_3_/C_3_	R_4_/C_4_	R_5_/C_5_	R_6_/C_6_	R_7_/C_7_
**2 Ω** 1 Ω ∠ 1 Ω	**300 kΩ** 150 kΩ ∠ 150 kΩ	**50 kΩ** 100 kΩ || 100 kΩ	**9 kΩ** 18 kΩ || 18 kΩ	**1.7 kΩ** 1 kΩ ∠ 680 Ω	**305 Ω** 270 Ω ∠ 33 Ω	**54 Ω** 27 Ω ∠ 27 Ω	**10 Ω** 10 Ω
**127 nF** 100 nF || 27 nF	**180 nF** 180 nF	**118 nF** 100 nF || 18 nF	**76 nF** 68 nF || 8.2 nF	**45.7 nF** 39 nF || 6.8 nF	**27.9 nF** 18 nF || 10 nF	**17 nF** 10 nF || 6.8 nF	**10.4 nF** 10 nF || 390 pF

**Table 33:** Approximation of CPE with order α = 4/5; RC series-parallel topology of fractal capacitor.

Rp/Cp	R_1_/C_1_	R_2_/C_2_	R_3_/C_3_	R_4_/C_4_	R_5_/C_5_	R_6_/C_6_	R_7_/C_7_
**940 kΩ** 820 kΩ ∠ 120 kΩ	**211 kΩ** 180 kΩ ∠ 33 kΩ	**38.7 kΩ** 33 kΩ ∠ 5.6 kΩ	**7.1 kΩ** 5.6 kΩ ∠ 1.5 kΩ	**1.3 kΩ** 1.2 kΩ ∠ 100 Ω	**239 Ω** 220 Ω ∠ 18 Ω	**44 Ω** 22 Ω ∠ 22 Ω	**7.5 Ω** 15 Ω || 15 Ω
**35.2 nF** 33 nF || 2.2 nF	**237 nF** 220 nF || 18 nF	**155 nF** 100 nF || 56 nF	**101 nF** 100 nF || 1 nF	**66.4 nF** 56 nF || 10 nF	**43.4 nF** 39 nF || 4.7 nF	**28.4 nF** 27 nF || 1.5 nF	**22 nF** 22 nF

**Table 34:** CPE with math order α = 4/5; fully passive RC parallel-series topology of fractal capacitor.

Rs/Cs	R_1_/C_1_	R_2_/C_2_	R_3_/C_3_	R_4_/C_4_	R_5_/C_5_	R_6_/C_6_	R_7_/C_7_
**4.3 Ω** 3.3 Ω ∠ 1 Ω	**470 kΩ** 470 kΩ	**91.7 kΩ** 82 kΩ ∠ 10 kΩ	**16.8 kΩ** 10 kΩ ∠ 6.8 kΩ	**3080 Ω** 2.7 kΩ ∠ 390 Ω	**565 Ω** 470 Ω ∠ 100 Ω	**104 Ω** 100 Ω ∠ 3.9 Ω	**19 Ω** 18 Ω ∠ 1 Ω
**52.8 nF** 47 nF || 5.6 nF	**90 nF** 180 nF ∠ 180 nF	**65.4 nF** 47 nF || 18 nF	**42.8 nF** 39 nF || 3.9 nF	**28 nF** 27 nF || 1 nF	**18.3 nF** 10 nF || 8.2 nF	**12 nF** 12 nF	**8.2 nF** 8.2 nF

**Table 35:** Approximation of CPE with order α = 8/9; RC series-parallel topology of fractal capacitor.

Rp/Cp	R_1_/C_1_	R_2_/C_2_	R_3_/C_3_	R_4_/C_4_	R_5_/C_5_	R_6_/C_6_	R_7_/C_7_
**10 MΩ** 10 MΩ	**1.6 MΩ** 1.5 MΩ ∠ 100 kΩ	**225 kΩ** 220 kΩ ∠ 4.7 kΩ	**31.4 kΩ** 680 kΩ || 33 kΩ	**4375 Ω** 3.9 kΩ ∠ 470 Ω	**611 Ω** 390 Ω ∠ 220 Ω	**85 Ω** 82 Ω ∠ 3.3 Ω	**12 Ω** 12 Ω
**20.3 nF** 18 nF || 2.2 nF	**23 nF** 22 nF || 1 nF	**19.4 nF** 18 nF || 1.5 nF	**15.2 nF** 13 nF || 2.2 nF	**12 nF** 12 nF	**9.3 nF** 4.7 nF || 4.7 nF	**7.3 nF** 4.7 nF || 2.7 nF	**6.2 nF** 4.7 nF || 1.5 nF

**Table 36:** CPE with math order α = 8/9; fully passive RC parallel-series topology of fractal capacitor.

Rs/Cs	R_1_/C_1_	R_2_/C_2_	R_3_/C_3_	R_4_/C_4_	R_5_/C_5_	R_6_/C_6_	R_7_/C_7_
**220 mΩ** 220 mΩ	**200 kΩ** 100 kΩ ∠ 100 kΩ	**27 kΩ** 27 kΩ	**3890 Ω** 3.9 kΩ	**543 Ω** 390 Ω ∠ 150 Ω	**76 Ω** 150 Ω || 150 Ω	**10.6 Ω** 6.8 Ω ∠ 3.9 Ω	**1.5 Ω** 1.5 Ω
**56 nF** 56 nF	**180 nF** 180 nF	**156 nF** 100 nF || 56 nF	**122 nF** 100 nF || 22 nF	**95.6 nF** 470 nF ∠ 120 nF	**74.7 nF** 68 nF || 6.8 nF	**58 nF** 56 nF || 2.2 nF	**45.7 nF** 39 nF || 6.8 nF

**Table 37:** Approximation of CPE with order α = 9/10; RC series-parallel topology of fractal capacitor.

Rp/Cp	R_1_/C_1_	R_2_/C_2_	R_3_/C_3_	R_4_/C_4_	R_5_/C_5_	R_6_/C_6_	R_7_/C_7_
**17 MΩ** 10 MΩ ∠ 6.8 MΩ	**3 MΩ** 1.5 MΩ ∠ 1.5 MΩ	**441 kΩ** 220 kΩ ∠ 220 kΩ	**65.4 kΩ** 33 kΩ ∠ 33 kΩ	**9.7 kΩ** 8.2 kΩ ∠ 1.5 kΩ	**1.44 kΩ** 1.2 kΩ ∠ 220 Ω	**214 Ω** 180 Ω ∠ 33 Ω	**32 Ω** 33 Ω
**14.4 nF** 12 nF || 2.2 nF	**12 nF** 12 nF	**9.8 nF** 8.2 nF || 1.5 nF	**7.9 nF** 6.8 nF || 1 nF	**6.4 nF** 5.6 nF || 820 pF	**5.2 nF** 3.3 nF || 1.8 nF	**4.2 nF** 3.9 nF || 330 pF	**3.7 nF** 3.3 nF || 390 pF

**Table 38:** CPE with math order α = 9/10; fully passive RC parallel-series topology of fractal capacitor.

Rs/Cs	R_1_/C_1_	R_2_/C_2_	R_3_/C_3_	R_4_/C_4_	R_5_/C_5_	R_6_/C_6_	R_7_/C_7_
**33 mΩ** 33 mΩ	**180 kΩ** 180 kΩ	**27 kΩ** 27 kΩ	**4 kΩ** 3.9 kΩ ∠ 100 Ω	**588 Ω** 560 Ω ∠ 27 Ω	**87 Ω** 82 Ω ∠ 4.7 Ω	**13 Ω** 12 Ω ∠ 1 Ω	**2.2 Ω** 2.2 Ω
**47 nF** 47 nF	**170 nF** 150 nF || 22 nF	**160 nF** 150 nF || 10 nF	**131 nF** 120 nF || 10 nF	**106 nF** 100 nF || 5.6 nF	**85.6 nF** 82 nF || 3.3 nF	**69.3 nF** 68 nF || 1.2 nF	**47 nF** 47 nF
